# Lack of the human choline transporter‐like protein SLC44A2 causes hearing impairment and a rare red blood phenotype

**DOI:** 10.15252/emmm.202216320

**Published:** 2023-01-25

**Authors:** Bérengère Koehl, Cédric Vrignaud, Mahmoud Mikdar, Thankam S Nair, Lucy Yang, Seyve Landry, Guy Laiguillon, Claudine Giroux‐Lathuile, Sophie Anselme‐Martin, Hanane El Kenz, Olivier Hermine, Narla Mohandas, Jean Pierre Cartron, Yves Colin, Olivier Detante, Raphaël Marlu, Caroline Le Van Kim, Thomas E Carey, Slim Azouzi, Thierry Peyrard

**Affiliations:** ^1^ Université Paris Cité and Université des Antilles, INSERM, BIGR Paris France; ^2^ Department of Child Hematology Reference Center for Sickle‐Cell Disease Robert Debré University Hospital, Assistance Publique‐Hôpitaux de Paris Paris France; ^3^ Kresge Hearing Research Institute, Department of Otolaryngology/Head and Neck Surgery University of Michigan Ann Arbor MI USA; ^4^ Hemostasis Laboratory Grenoble Alpes University Hospital Grenoble Grenoble France; ^5^ Établissement Français de Sang (EFS) Ile‐de‐France, Centre National de Référence pour les Groupes Sanguins Paris France; ^6^ Etablissement Français du Sang Auvergne Rhône Alpes, Immunohematology Laboratory Grenoble France; ^7^ Department of Transfusion, Blood Bank, CHU‐Brugmann and Hôpital Universitaire des Enfants Reine Fabiola Université Libre de Bruxelles Brussels Belgium; ^8^ Université de Paris, Imagine Institute, INSERM UMR 1163 Paris France; ^9^ Red Cell Physiology Laboratory, New York Blood Center New York NY USA; ^10^ Stroke Unit, Neurology Department, Grenoble Hospital, Grenoble Institute of Neurosciences, Inserm U1216 University of Grenoble Alpes Grenoble France; ^11^ University Grenoble Alpes, CNRS UMR5525, TIMC Grenoble France

**Keywords:** blood group antigen, hearing impairment, red blood cells, SLC44A2, transfusion, Genetics, Gene Therapy & Genetic Disease, Haematology, Vascular Biology & Angiogenesis

## Abstract

Blood phenotypes are defined by the presence or absence of specific blood group antigens at the red blood cell (RBC) surface, due to genetic polymorphisms among individuals. The recent development of genomic and proteomic approaches enabled the characterization of several enigmatic antigens. The choline transporter‐like protein CTL2 encoded by the *SLC44A2* gene plays an important role in platelet aggregation and neutrophil activation. By investigating alloantibodies to a high‐prevalence antigen of unknown specificity, found in patients with a rare blood type, we showed that SLC44A2 is also expressed in RBCs and carries a new blood group system. Furthermore, we identified three siblings homozygous for a large deletion in *SLC44A2*, resulting in complete SLC44A2 deficiency. Interestingly, the first‐ever reported SLC44A2‐deficient individuals suffer from progressive hearing impairment, recurrent arterial aneurysms, and epilepsy. Furthermore, SLC44A2_null_ individuals showed no significant platelet aggregation changes and do not suffer from any apparent hematological disorders. Overall, our findings confirm the function of SLC44A2 in hearing preservation and provide new insights into the possible role of this protein in maintaining cerebrovascular homeostasis.

## Graphical abstract

Homozygous deletion in the SLC44A2 gene was identified in three siblings with a rare blood phenotype. SLC44A2 was confirmed to be essential in the physiology of hearing in humans, but dispensable for erythropoiesis and platelet aggregation.

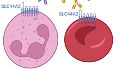

## Introduction

Blood group antigens are defined by the presence or absence of specific antigens on the surface of the red blood cell (RBC) membrane and are inherited characteristics resulting from genetic polymorphism at the identified blood group loci (Storry *et al*, 2019). A null RBC phenotype, in which all antigens in one system are absent, has been identified in most blood group systems, generally because of the complete absence of the antigen carrier molecule from the RBC membrane. In many cases, individuals with these null phenotypes are apparently healthy, suggesting that the biological function of the missing protein may be compensated by another mechanism (Helias *et al*, 2012; Saison *et al*, 2012; Azouzi *et al*, 2020; Mikdar *et al*, 2021). However, in some cases, the null phenotype is associated with mild‐to‐severe hematological and/or nonhematological disorders (Duval *et al*, 2021). Therefore, null phenotypes represent natural “knockouts” and represent unique opportunities in providing indications toward the function of membrane proteins, not only in erythroid cells but also in other cells or tissues (Bertelson *et al*, 1988; Preston *et al*, 1994; Cherif‐Zahar *et al*, 1996; Ribeiro *et al*, 2000; Azouzi *et al*, 2013; Daniels *et al*, 2015).

SLC44A2, also known as the choline transporter‐like protein 2 (CTL2) family, is present in various human tissues including kidney, lung, inner ear, endothelial, and blood cells. Currently, the molecular and cellular function of SLC44A2 is not well‐defined. In murine models, its deficiency has been associated with hearing loss, altered neutrophil recruitment, and impaired platelet activation (Bennett *et al*, 2020; Zirka *et al*, 2021). In humans, *SLC44A2* has been identified as a susceptibility locus for venous thromboembolism (VTE), which generated heightened interest in its function and attention has shifted to its role in thrombosis (Germain *et al*, 2015; Hinds *et al*, 2016). Several studies have recently confirmed that SLC44A2 promotes thrombosis in a mouse model of laser injury and venous stenosis and suggest that this may be related to platelet–neutrophil interaction (Tilburg *et al*, 2020). Accordingly, a recent study identified activated α_IIb_β_3_ integrin in platelets as a receptor and agonist for neutrophils through SLC44A2 (Constantinescu‐Bercu *et al*, 2020). In addition, a murine study showed that SLC44A2 is a mitochondrial choline transporter that regulates mitochondrial synthesis of ATP and platelet activation (Bennett *et al*, 2020). The mitochondrial function of this protein was also reported in the human brain microvascular endothelial cells of the blood–brain barrier. It has been proposed that SLC44A2 is responsible for choline transport into mitochondria, an important step in the oxidative pathway of choline metabolism (Iwao *et al*, 2016; Inazu, 2019).

Herein, we show that SLC44A2 is expressed in erythrocytes and carries new blood group antigens called RIF and VER, establishing the 39^th^ blood group system recognized by the International Society of Blood Transfusion (ISBT), the so‐called CTL2. Interestingly, we demonstrate that a large homozygous deletion in *SLC44A2* gene results in the total absence of this protein in three siblings, all of whom suffer from age‐related hearing loss, and some of them from epilepsy or intracranial aneurysms. Unexpectedly, the absence of SLC44A2 from platelets and RBCs does not cause any apparent hematological disorder. Finally, we demonstrated that those two new anti‐SLC44A2 red cell alloantibodies lead to neutrophil activation and their adhesion on endothelial cells.

## Results

### A single nucleotide variation in the *SLC44A2* gene encodes a new blood group antigen

**Figure 1:**
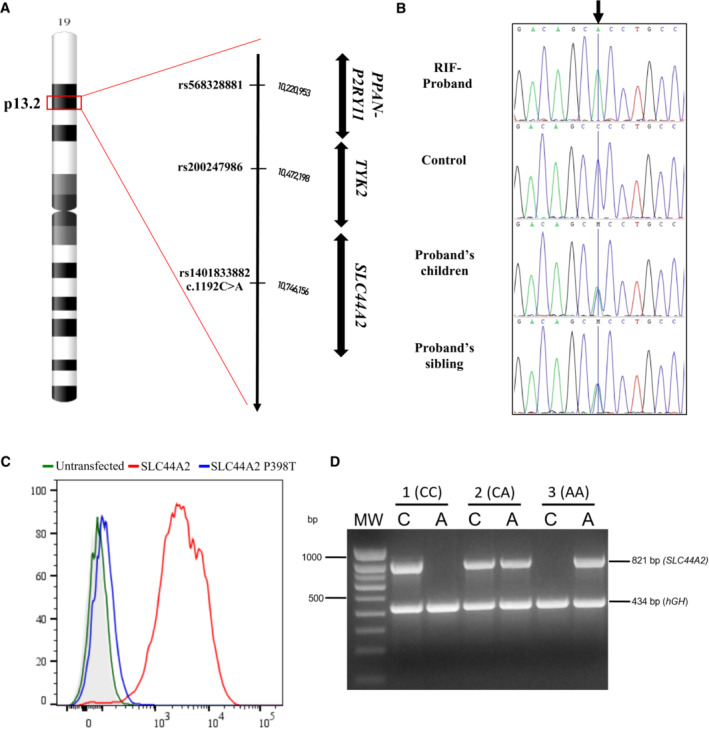
Molecular basis of the RIF blood group antigen Common variant candidates found in RIF^−^ North African individuals were shown in an ideogram of chromosome 19, highlighting a region of 19p13.2 with a red box. An expanded view of this region is shown, including the genes and chromosomal coordinates.Sequencing chromatograms of the *SLC44A2* variant in proband 1's family. Proband 1's child and sibling are heterozygous, whereas the RIF^−^ proband is homozygous for the c.1192C > A mutation (noted by the black arrow).Exogenous expression of SLC44A2 in L‐929 cells results in cell surface expression of the RIF antigen. L‐929 cells stably transfected with pcDNA3.1/SLC44A2 full‐length cDNA (red) or with cDNA3.1/SLC44A2(1192A) (blue) and then analyzed by flow cytometry with anti‐RIF antibody. The green profile corresponds to untransfected L‐929 (wild‐type), and the light gray profile corresponds to wild‐type L‐929 cells incubated with only the secondary antibody.Representative genotyping of the c.1192C > A mutation by allele‐specific PCR. For each sample, two PCRs were performed, one with a specific primer for the 1192C allele (PCR 1) and a second with specific primers for the 1192A allele (PCR 2). An 821‐bp band indicated the presence of the allele; amplification failure indicated the absence of the allele. The 434 bp amplification product of the *hGH* control primer is presented in all lanes. In the gel, the first lane (MW) shows the 100 bp DNA ladder marker (Eurogentec, Belgium); for the other lanes, C and A indicate the product from PCR 1 and PCR 2, respectively, amplified from RIF^+/+^ (1(CC)), RIF^+/−^ (2(CA)) and RIF^−/−^ (3(AA)) samples.

As part of an effort to identify the genes encoding high‐prevalence blood group antigens with an unknown molecular basis, we focused here our research on the serum of a pregnant woman, proband 1, born in Morocco (Rif coast area), who developed an RBC antibody during her pregnancy. A multilaboratory investigation was initially inconclusive, and the antibody was claimed to be of unknown specificity. We propose calling this antibody anti‐RIF and the new high‐prevalence antigen RIF. Further testing of this antibody with several RBC samples lacking a high‐prevalence antigen of unknown specificity allowed the identification of six more RIF^−^ samples from five North African and one individual of European descent. To identify the gene underlying the RIF antigen, we performed whole‐exome sequencing (WES) of these seven RIF^−^ individuals. After analysis of WES data, three shared variants (with minor allele frequency, MAF < 1%) were found in the six RIF^−^ North African individuals but not in the European proband: one in *SLC44A2* (c.1192C > A [p.Pro398Thr] [GenBank: NM_001145056]), a second in *PPAN‐P2RY11* (c.822 + 31 T > C [GenBank: NM_020230]), and a third in *TYK2* (c.2036G > C. [p.Arg679Pro] [GenBank: NM_003331]) genes. These three genes are located on chromosome 19p13.2 (Fig 1A). The *SLC44A2*, *PPAN‐P2RY11*, and *TYK2* variants are all present in the Genome Aggregation Database (gnomAD) browser, but the MAF values are available only for *PPAN‐P2RY11* (0.00003) and *TYK2* (0.0002). *SLC44A2* was considered the most prominent candidate because this gene encodes the choline transporter‐like 2 (CTL2), a membrane protein expressed in blood cells (Greinacher *et al*, 2010; Flesch *et al*, 2013; Bryk & Wisniewski, 2017; Tilburg *et al*, 2018). Family segregation confirmed that proband 1 (RIF^−^) was homozygous for these variants, while her children and sibling were heterozygous for the *SLC44A2* variant (RIF^−^/RIF^+^ phenotype; Fig 1B). To determine whether the *SLC44A2* polymorphism is responsible for the RIF^−^ blood type, we transfected murine L‐929 cells with an *SLC44A2* expression vector and analyzed them by flow cytometry using the anti‐RIF antisera. Exogenous expression of *SLC44A2* in L‐929 cells was responsible for cell surface expression of the RIF antigen. By contrast, the anti‐RIF antibody failed to bind the L‐929 cells expressing the p.Pro398Thr mutant of *SLC44A2* encoded by the c.1192C > A variant found in the RIF^−^ proband 1 (Fig 1C), indicating that Pro398 is important for the expression of the RIF antigen and contributes to the epitope recognized by the anti‐RIF. Altogether, these results demonstrated that the RIF^−^ phenotype results from the p.Pro398Thr substitution in SLC44A2. This finding allowed us to develop a genotyping assay to identify rare RIF^−^ blood donors. An AS‐PCR assay for the c.1192C > A variant (Fig 1D) confirmed the homozygous and heterozygous state of the *SLC44A2* mutation in the proband and her sibling, respectively, in accordance with their RIF phenotype (Appendix Fig S1).

### A large deletion in the *SLC44A2* gene underlies a null phenotype

**Figure 2:**
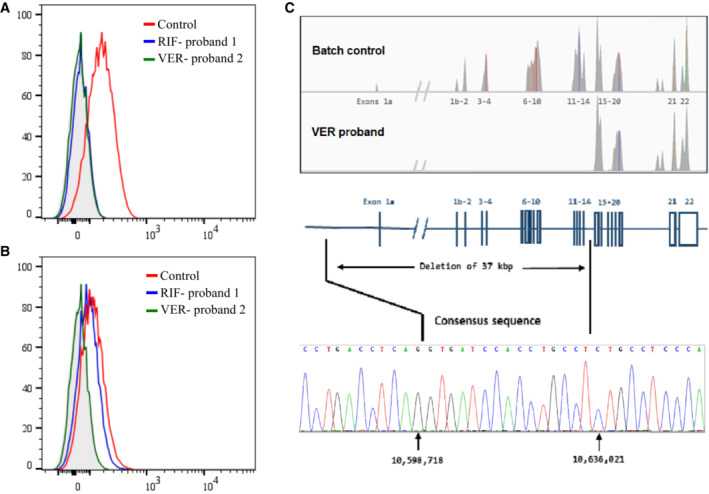
Null allele of *SLC44A2* is responsible for the rare VER^−^ blood type A, BFlow analysis of SLC44A2 expression at the surface of RBCs from control donors (red), RIF^−^ proband 1 (blue), and VER^−^ proband 2 (green) using anti‐RIF (A) and anti‐VER antibodies (B). The light gray profile corresponds to RBCs incubated with only the secondary antibody.CIntegrative Genomics Viewer analysis reveals homozygous deletion of exons from 1 to 14 of the *SLC44A2* gene in VER^−^ proband. Schematic representation of the *SLC44A2* gene and Sanger sequencing confirmation of the deletion breakpoints in genomic DNA of VER^−^ proband.

**Figure 3:**
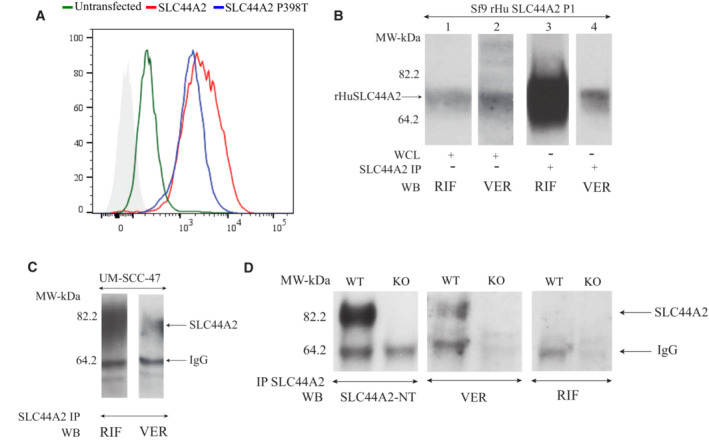
Reactivity of VER and RIF antibodies Exogenous expression of SLC44A2 in L‐929 cells results in cell surface expression of the VER antigen. L‐929 cells stably transfected with pcDNA3.1/SLC44A2 full‐length cDNA (red) or with cDNA3.1/SLC44A2(1192A) (blue) and then analyzed by flow cytometry with anti‐VER antibody. The green profile corresponds to untransfected L‐929 (wild‐type), and the light gray profile corresponds to wild‐type cells incubated with only the secondary antibody.Western blotting shows that the anti‐RIF and anti‐VER antibodies bind to HuSLC44A2. Western blots of whole Sf9 lysates (WCL) expressing full‐length recombinant human *SLC44A2* cDNA (rHuSLC44A2) were probed with RIF and VER antisera (1:10). Lanes 1 and 2 show strong binding to rHuSLC44A2. Titer differences in the sera required longer exposure (10 min) of the filter to the film to develop the image with VER serum compared with only 30 s exposure for RIF serum. A similar blot of immunoprecipitated rHuSLC44A2 from the Sf9 lysates similarly reacted with RIF and VER sera (lanes 3 and 4) showed equivalent exposure times (30 s for both sera) when tested against the concentrated rHuSLC44A2 protein.The anti‐RIF and VER antibodies bound to immunoprecipitated wild‐type SLC44A2 in lysates of the UM‐SCC‐47 human squamous cancer cell line (exposure time 5 min).Western blot of murine Slc44a2 protein immunoprecipitated from wild‐type and *Slc44a2* knockout mice with rabbit anti CTL2‐NT serum was probed with anti CTL2‐NT (left panel) and with VER serum (center Panel) or RIF serum (right panel). The left panel was developed with goat anti rabbit IgG heavy and light chain (Bethyl Lab cat # A120‐113P); the center and right panels were developed with rabbit anti human IgG and IgM heavy and light chain specific (Jackson Immunochemicals cat # 309035107).
Source data are available online for this figure.

whole‐exome sequencing data from the RIF^−^ European proband 2 indicated the absence of the c.1192C > A mutation in the *SLC44A2* gene despite her RBCs not reacting with anti‐RIF antibody (Fig 2A). To further characterize the blood phenotype of this proband, we first performed additional serological investigation by testing her RBC antibody onto the RBCs of a RIF^−^ North African individual. Flow cytometry experiments showed that this alloantibody was not an anti‐RIF because it reacted with RIF^−^ RBCs (Fig 2B). We proposed to call this antibody anti‐VER after the city of birth of proband 2 (Verona, Italy). Furthermore, the fact that this antibody was developed by a RIF^−^ proband but was not an anti‐RIF suggests that this European proband does not exhibit the same molecular basis as the North African individuals. Next, we assessed the coverage of the 22 exons of *SLC44A2* by analyzing copy‐number variation (CNV) data and identified a putative homozygous intragenic deletion in *SLC44A2*, which completely removed the first 15 exons along with the 5′ UTR region (Fig 2C). The exons involved in this large deletion encode the first 410 amino acids of SLC44A2 protein, which include the Pro398 involved in the expression of the RIF antigen. Using a primer walking approach and Sanger sequencing, the precise break points of the deletion in the intergenic region and intron 14 were identified, with coordinates from chromosome 19:10598733‐10636021 (GRCh38/hg38 assembly; Fig 2C). This deletion defines a null allele of *SLC44A2*, which could explain the rare blood phenotype of proband 2. Furthermore, flow cytometry analysis of L‐929 cells stably transfected with the *SLC44A2* cDNA showed cell surface expression of the VER antigen, confirming that the anti‐VER antibody is directed against the SLC44A2 protein (Fig 3A). We then tested the binding of antibodies from RIF^−^ individuals and from SLC44A2_null_ proband 2 (VER^−^) on recombinant human SLC44A2 protein (rHuSLC44A2) produced in transfected Sf9 insect cells (Kommareddi *et al*, 2009). rHuSLC44A2, produced in Sf9 cells either from whole cell lysates or immunoprecipitated with rabbit anti‐SLC44A2 (CTL2‐NT; Nair *et al*, 2004; Kommareddi *et al*, 2010), was used to assess serum alloantibodies from probands 1 and 2. As shown in Fig 3B, both RIF and VER antibodies reacted specifically with whole cell lysates or with rHuSLC44A2 concentrated by immunoprecipitation. Serum from proband 1 exhibited a higher titer and required only a 30‐s exposure of the ECL blot to show binding to the rHuSLC44A2 protein in Sf9 whole cell lysates (WCL) (Fig 3B, lane 1), whereas the proband 2 serum required a 10‐min exposure to reach the same saturation (Fig 3B, lane 2). By contrast, when the rHuSLC44A2 protein was concentrated by immunoprecipitation, binding was detectable in 30 s with both anti‐VER and anti‐RIF sera (Fig 3B, lanes 3 and 4). Both sera also reacted against immunoprecipitated SLC44A2 from a human squamous cancer cell line (UM‐SCC‐47) that expresses high levels of the protein (Fig 3C, lanes 1 and 2). In another approach, the alloantibodies from proband 1 and 2 were also tested against cell extracts of wild‐type and *Slc44a2* knockout mice (Kommareddi *et al*, 2015). The VER serum, which arose in SLC44A2_null_ proband 2, binds well to the wild‐type mouse lung Slc44a2 protein but not to the knockout murine lung protein, supporting the specificity of the alloantibodies for SLC44A2. A review of the mouse amino acid sequences showed that most of the murine Slc44A2 protein is homologous to the human form, but the region carrying the RIF antigen in humans was dissimilar in the mouse. The mouse lacks c.1192proline and has alanine in that site, explaining why anti‐RIF fails to bind to the wild‐type mouse protein (Fig 3D, right panel; amino acid sequence comparison in Appendix Fig S2).

### Null allele of *SLC44A2* is associated with hearing loss

**Figure 4:**
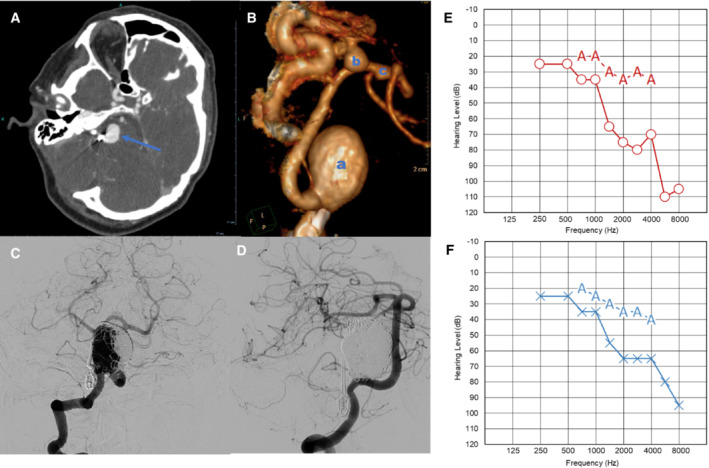
Clinical features of the VER^−^ proband ACT angiography of the giant unruptured intracranial aneurysm on the right posterior cerebellar artery (PCA, blue arrow).B3D volume rendered of multiple cerebral aneurysms on the right PCA (a), on right posterior communicating artery (b), and on right middle cerebral artery (c).C, DAngiograms before and after embolization of the PCA aneurysm, respectively.E, FAudiograms of VER‐ proband, showing a hearing loss by air conduction audiometry (blue line) and bone conduction audiometry (red line) for both left and right ear, witnessing a perception hearing loss in this patient.

Proband 2, a European in their 60s homozygous for a null *SLC44A2* allele, was the first‐ever reported individual with no expression of SLC44A2. Clinically, she suffered from major and unusual cerebrovascular anomalies that started in her 30s. She showed a giant intracranial aneurysm (ICA) of the right posterior cerebellar artery treated by neurosurgery and by endovascular embolization (Fig 4A, C and D). Shortly after, she also developed a right posterior communicating artery ICA treated by endovascular embolization and a small asymptomatic aneurysm on the right middle cerebral artery (Fig 4B). She later died from a hemorrhagic stroke. In addition, proband 2 was also diagnosed with hearing impairment, requiring a hearing aid device a few years before her death. Otoscopic examination of the ear was normal, but the audiometry tests revealed bilateral perceptive hearing impairment in the upper frequency range (Fig 4E and F). Two of the proband's siblings were homozygous for the *SLC44A2* deletion and are phenotyped as VER^−^, and one of them (IV.4 in Appendix Fig S3) developed an anti‐VER alloantibody after RBC transfusion. Clinically, both siblings developed epileptic seizures and hearing loss from their 30s. The VER^+^ siblings with a heterozygous deletion of *SLC44A2* only suffer from hearing loss, while the wild‐type VER^+^ sibling (IV.7 in Appendix Fig S3) is healthy. In accordance with *Slc44a2* ^−/−^ and *Slc44a2* ^+/−^ mice, the *SLC44A2* null allele in proband 2 and her siblings is associated with progressive age‐related hearing impairment (Kommareddi *et al*, 2015). Interestingly, in the proband 2 family, the homozygous *SLC44A2* null allele was associated with more severe diseases, including ICA and epilepsy.

### SLC44A2 is dispensable for erythropoiesis and platelet aggregation

**Figure 5:**
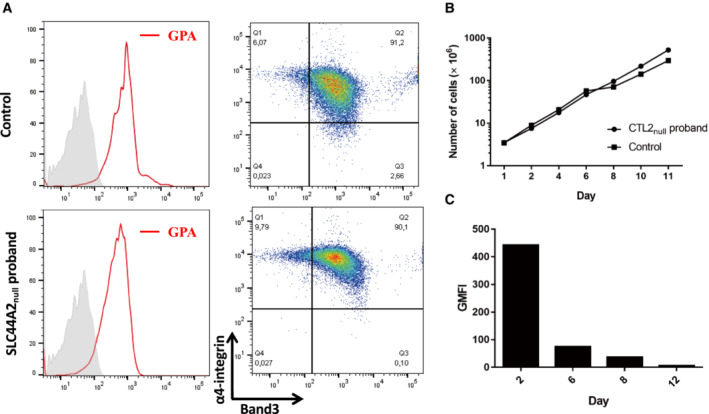
Normal *ex vivo* erythropoiesis of SLC44A2_null_ HSPCs Terminal differentiation of HSPCs from healthy control and SLC44A2_null_ proband was monitored via α4‐integrin and band 3 levels of GPA^pos^ cells at day 6 after EPO addition.Cell proliferation during *in vitro* erythropoiesis.SLC44A2 expression during terminal erythroid differentiation of HSPCs from healthy controls using an anti‐VER antibody.

Choline is an essential metabolite for cells because it plays an important role in the synthesis of the phospholipids that maintain membrane integrity. SLC44A2 was originally called CTL2 for choline transporter‐like protein 2 because it has homology to other choline transporters. It belongs to the same SLC family as CTL1 (SLC44A1), which was discovered to complement a choline transport deficient yeast strain using RNA from the electric organ of the Torpedo fish (O'Regan *et al*, 2000; O'Regan & Meunier, 2003). SLC44A2 in the cell membrane was found to be a poor choline transporter when compared to its homolog SLC44A1, and its function is still not well‐understood (Kommareddi *et al*, 2010). To better appreciate the function of SLC44A2 in erythroid cells, we analyzed the rheological properties of the RBCs from the SLC44A2_null_ proband 2. The proband 2 RBCs displayed normal RBC deformability (Appendix Fig S4), suggesting that the absence of SLC44A2 does not alter the biophysical properties of RBC membranes. Next, we compared the proliferation and differentiation of hematopoietic stem and progenitor cells (HSPCs) isolated from the peripheral blood of SLC44A2_null_ proband and from a healthy donor as a control. Markers used to assess the progression of erythroid differentiation included glycophorin A (GPA), band 3, and CD49d (α4 integrin) and showed normal *in vitro* erythroid proliferation and differentiation (Fig 5A and B), which is consistent with the absence of erythroid disorders in this proband and her VER^−^ sibling (Appendix Table S1). In addition, the monitoring of SLC44A2 expression during erythropoiesis from control HSCs using anti‐VER antibody showed a higher expression level in immature erythroblasts (pro‐erythroblast stage, Day 2) followed by a progressive strong decrease upon erythroid terminal maturation (Fig 5C). On the contrary, a recent study in *Slc44a2* ^−/−^ mice showed that Slc44a2 controls platelet activation via its function as a mitochondrial choline transporter (Bennett *et al*, 2020). Both SLC44A2_null_ patients (IV.1 and IV.5 in Appendix Fig S3) showed normal complete blood count parameters, compared with reference values, including platelet count (Appendix Table S1). To evaluate the role of SLC44A2 in human platelet activation, we assessed aggregation and activation characteristics of platelets from two SLC44A2_null_ individuals, in the presence of several agonists. In both cases, *ex vivo* platelet aggregation was normal in response to the thrombin receptor activating peptide (TRAP), arachidonic acid, ADP, ristocetin, and collagen at 5 μM (Table 1). Patient IV.5 showed a defect of aggregation in the presence of epinephrine even at high concentration (25 μM), whereas his sister had a strictly normal response at low concentration (5 μM). By itself, the lack of response to epinephrine is not pathological since some healthy individuals may also not respond to epinephrine without a clinically significant platelet defect (Scrutton *et al*, 1981). Furthermore, platelet activation was assessed using flow cytometry analysis of CD62P (P‐selectin) and CD63 expression. In the presence of different agonists, SLC44A2_null_ and control platelets have similar levels of P‐selectin and dense granule release by CD63 translocation (Appendix Fig S5). It is noteworthy that the proband 2, as well as the 2 others homozygous SLC44A2_null_ probands, never exhibited any coagulation disorder such as frequent bleeding (epistaxis, profuse menstruation, gingivorrhagia, etc). In summary, we showed that human SLC44A2 is dispensable for *ex vivo* erythropoiesis and platelet aggregation.

**Table 1:** Aggregation tests of platelets from healthy donors and SLC44A2_null_ individuals.

	CTL2null (IV.1 patient)		CTL2null (IV.5 patient)		Healthy donor 1		Healthy donor 2	
	Max aggregation (%)	Slope (%/min)	Max aggregation (%)	Slope (%/min)	Max aggregation (%)	Slope (%/min)	Max aggregation (%)	Slope (%/min)
Collagen (2 μg/ml)	24	31	3	7	78	58	73	60
Collagen (5 μg/ml)	67	77	80	87	76	79	79	92
ADP (5 mM)	75	100	65	77	79	100	80	97
Arachidonic acid (1 mM)	92	78	85	71	80	78	85	86
Epinephrine (5 μM)	84	20	12	11	77	44	90	40
Epinephrine (25 μM)	–	–	28	27	–	–	80	44
TRAP (25 μM)	81	109	86	112	81	111	89	108
Ristocetin (1 mg/ml)	87	64	85	63	89	116	78	50

### Anti‐SLC44A2 alloantibodies activate neutrophil adhesion to endothelial cells

**Figure 6:**
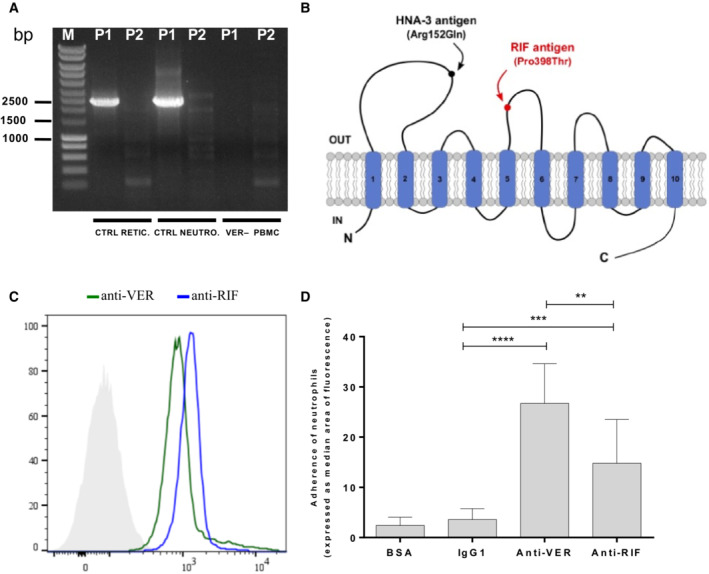
Anti‐SLC44A2 red cell alloantibodies activate neutrophil adhesion to endothelial cells SLC44A2 transcript variants P1 and P2 were amplified from cDNA samples isolated from control reticulocytes (CTRL RETIC), neutrophils (CTRL NEUTRO), and PBMC of the VER^−^ proband 2 (VER PBMC). The purity of reticulocytes and neutrophils was verified by flow cytometry using anti‐CD71 and anti‐CD16 antibodies, respectively.Predicted topology of the human SLC44A2 (isoform P1). The location of the HNA‐3 (Arg152Gln) and RIF (Pro398Thr) antigens are highlighted. Transmembrane domains are numbered 1–10. The P1 transcript variant encodes the shorter isoform of 704 amino acids in which arginine determining the HNA‐3 antigen is at position 152.Flow cytometry analysis of neutrophil cells from control donors with anti‐RIF (blue) and anti‐VER (green) antibodies. The gray profile corresponds to neutrophils incubated with the secondary antibody only.Effect of anti‐VER and anti‐RIF on the adhesion of neutrophils from healthy controls to endothelial cells (HMEC‐1). Adherent neutrophils were quantified and means ± standard error of the median of three independent experiments are shown. **P < 0.01, ***P < 0.001, ****P < 0.0001. Statistical analyses were performed using GraphPad Prism 7. Statistical test was performed using paired nonparametric *t*‐test (Wilcoxon *t*‐test).

SLC44A2 protein is highly expressed on neutrophils and carries the human neutrophil antigen‐3 (HNA‐3; Greinacher *et al*, 2010). Given that SLC44A2 is expressed in two isoforms (P1 and P2 transcripts), we compared the SLC44A2‐specific cDNA derived from neutrophils and reticulocytes. We showed by RT‐PCR using specific primers for both isoforms that reticulocytes expressed only the P1 variant, such as neutrophils (Fig 6A). The absence of P1 and P2 transcripts in the PBMCs from the VER^−^ proband 2 confirmed her null phenotype. Anti‐HNA‐3 antibodies were reported to be frequently involved in transfusion‐related acute lung injury (TRALI) through neutrophil activation and aggregation in the pulmonary microvasculature. The human neutrophil antigen 3a (HNA‐3a) is located in the first loop of SLC44A2 and is the result of a nonsynonymous single nucleotide polymorphism leading to an amino acid exchange p. Arg152 > Gln (isoform P1)/p.Arg154 > Gln (isoform P2; Fig 6B). We next tested whether anti‐VER and anti‐RIF antibodies were reactive on neutrophils. Flow cytometry data showed that both antibodies recognized SLC44A2 protein in neutrophils (Fig 6C). To investigate the effect of these antibodies on neutrophil adhesion, neutrophils from healthy donors were isolated and incubated with both antibodies and then perfused through channels coated with endothelial cells, HMEC‐1 (*n* = 3). After 45 min of perfusion, we quantified the neutrophils and the neutrophils aggregates adhering to the endothelial cells. Both anti‐VER and anti‐RIF alloantibodies significantly increase neutrophil adhesion to endothelial cells, as well as neutrophil aggregate formation compared with nonimmune human IgG1 (*P* < 0.0001 and *P* < 0.001, respectively). This result suggested that anti‐VER and anti‐RIF antibodies may induce aggregates of neutrophils, like that caused by anti‐HNA‐3 antibodies.

## Discussion

Here, we provide convincing evidence for the identification of a novel blood group system, as defined by two human RBC alloantibodies (anti‐VER and anti‐RIF), encoded by the *SLC44A2* gene located on chromosome 19p13.2. The gene product is the SLC44A2 protein (choline transporter‐like protein 2), which carries two antigens as of today, the RIF and VER high‐prevalence antigens. The rare RIF^−^ blood type, found in several Moroccan individuals with an anti‐RIF alloantibody, is caused by the p.Pro398Thr substitution in SLC44A2 protein. This result indicates that Proline 398, located in the third extracellular loop of SLC44A2 (Fig 6B), encodes the RIF antigen and that Threonine 398 is the molecular basis of the rare RIF^−^ phenotype. Since the large deletion in *SLC44A2* leads to the null VER^−^ phenotype (no SLC44A2 protein), the RIF antigen is consequently not expressed in VER^−^ RBCs (Fig 2A). Similarly, we have recently demonstrated that the Augustine antigen At^a^, carried by the Glutamine 391 in ENT1 nucleoside transporter, is absent from ENT1_null_ RBCs (AUG:‐1 phenotype; Daniels *et al*, 2015).

There was no history of hemolytic disease of the fetus and newborn, and acute hemolytic transfusion reaction in RIF^−^ and VER^−^ families, indicating that both antibodies are not clinically significant in RBC transfusion. However, these antibodies induce *ex vivo* neutrophil adhesion to endothelial cells, suggesting a potential implication within TRALI syndrome after plasma transfusion. TRALI is a life‐threatening complication and remains a leading cause of transfusion‐associated mortality. HNA‐3a antibodies are the most frequent detected of the HNA antibodies in TRALI cases (Storch *et al*, 2014). These antibodies were shown to directly prime neutrophils, resulting in the formation of NETs an enabling neutrophil‐mediated pulmonary endothelial cell damage (Silliman *et al*, 2007; Thomas *et al*, 2012).

In addition, our data confirm that SLC44A2 is expressed in human erythroid precursors and mature RBCs, but at a lower level compared with its expression on neutrophils (Figs 2B and 6C). The p.Pro398Thr variant found in RIF^−^ North African individuals is predicted to be nonpathogenic by the SIFT and PolyPhen software and does indeed not significantly affect the expression level of SLC44A2 on RBCs (Fig 2B). However, the absence of erythroid defects in proband 2 and her sibling (IV.5 in Appendix Fig S3), as well as normal *in vitro* erythropoiesis, indicate that SLC44A2 is dispensable for normal erythroid proliferation and differentiation. In neutrophils, SLC44A2 is highly expressed (~ 5 × 10^5^ copies/cell; Rieckmann *et al*, 2017). However, we have no evidence that the SLC44A2_null_ phenotype is associated with innate immunity dysfunction, as SLC44A2_null_ individuals have no significant history of severe infection. Rather, its absence in the two SLC44A2_null_ probands has no consequence on the neutrophil count in accordance with the *slc44a2* ^*−/−*^ mice model (Appendix Table S1), suggesting that other choline transporter(s) may compensate for the absence of SLC44A2 in these cells. Considering platelet function, a recent study in mice showed that Slc44a2 (KO) platelets aggregate less than Slc44a2 (WT) platelets after thrombin treatment (Bennett *et al*, 2020). In other mice studies, platelet activation and aggregation under both static and shear conditions were normal for blood from *Slc44a2* ^*−/−*^ mice (Tilburg *et al*, 2018, 2020). Importantly, human SLC44A2_null_ platelets displayed normal aggregation with several agonists including TRAP at different concentrations. These results are consistent with the absence of thromboembolic complication in the three SLC44A2_null_ individuals of this study. We and others have shown that only P1 isoform is expressed in reticulocytes, neutrophils, and platelets (Kommareddi *et al*, 2010; Flesch *et al*, 2013). As only the P2 isoform is able to transport choline (Kommareddi *et al*, 2010), the biological function of the P1 isoform in these blood cells remains unclear. Collectively, the absence of hematological disorders in SLC44A2_null_ patients is particularly intriguing considering the reported function of this protein in a murine model. An attractive hypothesis to explain this difference may be related to a possible genetic compensation in SLC44A2_null_ patients.

Nevertheless, *SLC44A2* polymorphisms or mutations in humans are far from harmless. Indeed, *SLC44A2* polymorphisms have been identified in genome‐wide association studies to have an allele‐specific association with venous thromboembolism and cardiovascular diseases (Germain *et al*, 2015; Hinds *et al*, 2016). More recently, venous thromboembolism recurrence in Thai patients was shown to be linked to SLC44A2 p.Arg152Gln variant (Apipongrat *et al*, 2019). Considering our SLC44A2_null_ patient (proband 2), she suffered from multiple atypical ICAs, which characteristics (onset at a young age, large size, multiple sites, recurrence after treatment) are consistent with a congenital origin rather than a sporadic phenomenon (Krischek & Inoue, 2006; Alg *et al*, 2013; Caranci *et al*, 2013). In addition, the two other homozygous SLC44A2_null_ siblings were both reported as having both epileptic disease occurring between 30 and 40 years old, but we lack the cerebral investigations allowing us to determine the presence of ICA in these patients. Nevertheless, it is difficult to assess the significance of the association between SLC44A2_null_ phenotype and these pathologies based only on this family case. Further investigations in additional unrelated SLC44A2_null_ individuals are necessary to fully appreciate the relevance of this observation. However, the progressive hearing impairment related to the *SLC44A2* deletion in this family is consistent with the hearing deficiency observed in the *slc44a2* ^−/−^ mice, which was shown to be secondary to loss of auditory sensory cells, and spiral ganglion cells (Kommareddi *et al*, 2015). Our data strongly validate the crucial function of SLC44A2 in hearing maintenance.

In summary, our data demonstrate that defective alleles of *SLC44A2* occur in humans, do not preclude viability, and are not necessarily associated with hematological disorders observed in murine models. However, the precise role of SLC44A2 in cerebrovascular maintenance remains to be established in mice and humans, especially as this protein is highly expressed in the brain–blood barrier.

## Materials and Methods

### Subjects

Samples from subjects lacking a high‐prevalence antigen of unknown specificity were cryopreserved at the National Reference Center for Blood Groups (CNRGS, biocollection #DC‐2016‐2872). Informed consent for research studies was obtained for all subjects in accordance with the principles set out in the WMA Declaration of Helsinki and the Department of Health and Human Services Belmont Report. The study was approved by local institutional review boards.

### Clinical reports of the probands

Proband 1, a North African woman, was identified during her second pregnancy through a routine RBC antibody screen. Her serum was found to react 2+ with all RBCs tested, including a comprehensive panel of RBCs with a rare phenotype from the collections of the CNRGS, with the exception of her own RBCs. The antibody titration test was consistent with a so‐called “HTLA” (high titer low affinity) profile (titer 512, 2+ reactivity until dilution 1/256). At 37 weeks of gestation, the proband naturally gave birth to a healthy boy. We decided to name this antibody anti‐RIF for the RIF region of Northern Africa where she lives.

Proband 2, a European woman was hospitalized for intraparenchymal hemorrhage due to a fall. Her clinical history showed a giant cerebral aneurysm at the posterior inferior cerebellar region, treated by surgical clipping, followed by a plausible transfusion. Her serum was positive with a large panel of RBCs with a rare phenotype (negative autocontrols), with no possibility of finding an antibody specificity. We decided to name this antibody anti‐VER after the proband's city of birth.

### Blood group serology and subjects screening

Antibody identification and RBC typing were performed by indirect antiglobulin gel‐test (ID‐Card LISS/Coombs; DiaMed, Bio‐Rad). The serum of proband 1 containing anti‐RIF was used to screen compatible subjects from cryopreserved RBCs lacking a high‐prevalence antigen of unknown specificity.

### Exome sequencing and data analysis

Genomic DNA was extracted from whole blood cells using a benchtop instrument for fully automated nucleic acid purification (MagNaPure Compact, Roche Life Science) according to the manufacturer's recommendations. Exome capture was performed with the Sure Select Human All Exon Kit (Agilent Technologies). Agilent Sure Select Human All Exon (58 Mb, V6) libraries were prepared from 3 μg of genomic DNA sheared with an Ultrasonicator (Covaris) as recommended by the manufacturer. Barcoded exome libraries were pooled and sequenced with a HiSeq2500 system (Illumina), generating paired‐end reads. After demultiplexing, sequences were mapped on the human genome reference (NCBI build 37, hg19 version) with BWA. The mean depth of coverage obtained for RIF^−^ individuals exome libraries was >120X with >= 96% and >= 94% of the targeted exonic bases covered at least 15 and 30 independent sequencing reads. Variant calling was carried out with the Genome Analysis Toolkit (GATK), SAMtools, and Picard tools. Single nucleotide variants were called with GATK Unified Genotyper, whereas indel calls were made with the GATK IndelGenotyper_v2. All variants with read coverage 23% and Phred‐scaled quality 20% were filtered out with PolyWeb, an in‐house‐developed annotation software.

### Sanger sequencing

Exon 14 of the *SLC44A2* gene was amplified with primers SLC44A2‐P1F (forward) and SLC44A2‐P2R (reverse) from genomic DNA (Appendix Table S2). The PCR product was sequenced using the SLC44A2‐S1F primer. The region containing the deletion break points in the *SLC44A2* gene of the VER^−^ proband 2 were amplified with PCR primers SLC44A2‐P3F and SLC44A2‐P4R and sequenced using SLC44A2‐P2R. Detailed PCR conditions are available upon request.

### Genotyping

Genotyping of the RIF^−^ proband 1 family was performed in genomic DNA by allele‐specific PCR (AS‐PCR) analysis. Two PCRs were performed with specific reverse primers: SLC44‐1192C‐R for the 1192C allele, SLC44‐1192A‐R for the 1192A allele and a common forward primer SLC44A2‐P5F. The PCR products were interpreted on the basis of positive or negative amplification. As an internal control, two additional primers (hGH‐F and hGH‐R) for *hGH* amplification were used (Appendix Table S2).

### Erythroid cell culture and characterization

Peripheral blood mononuclear cells (PBMCs) were obtained from blood samples of healthy donors and VER^−^ proband 2 using Ficoll density gradient separation (Pancoll 1.077 g/ml, Pan‐Biotech). CD34^+^ cells were purified using anti‐CD34 conjugated microbeads and manual cell separation columns according to the manufacturer's instructions (Miltenyi). The cell culture procedure was comprised of two phases as described by Freyssinier *et al* (1999). SLC44A2 expression during terminal erythroid differentiation of HSCs from healthy controls was monitored by flow cytometry using an anti‐VER antibody.

### Plasmid construction

The pcDNA3.1/V5‐HisTOPO plasmid containing the full‐length *SLC44A2* cDNA (NM_001145056.1) was kindly provided by Sentot Santoso (Bayat *et al*, 2013). Plasmids with the c.1192A mutation were prepared using the QuikChange XL Site‐Directed Mutagenesis Kit (Stratagene) according to the manufacturer's protocol. A detailed mutagenesis PCR protocol is available upon request.

### Cell culture and transfection

L‐929 cells were grown in DMEM (Gibco) supplemented with 10% fetal bovine serum (Pan‐Biotech) and 0.5× antibiotic‐antimycotic solution (Gibco) at 37°C under a humidified atmosphere containing 5% CO_2_. Then, 3.10^5^ L‐929 cells were transfected with 1 μg of the plasmid using FuGENE® 6 Transfection Reagent as recommended by the manufacturer (Roche). Stable L‐929 transfectants were obtained after 3 weeks of selection with Geneticin (1 mg/ml, Thermo Fisher Scientific).

### Flow cytometry analyses in RBCs and L‐929 cells

Anti‐RIF and anti‐VER antibodies were purified by adsorption‐elution of sera from the RIF^−^ and VER^−^ probands, respectively, on papain‐treated RBCs using the Gamma Elu Kit II (Immucor). Thawed RBCs or fresh L‐929 cells were washed in Dulbecco's phosphate‐buffered saline solution (Gibco), resuspended in low‐ionic strength BFI buffer supplemented with 1% BSA and incubated with purified antibodies (1:2) at 37°C for 1 h. Anti‐RIF and anti‐VER labeling were revealed with goat F(ab′)2 anti‐human IgG (H + L)‐PE (1:50; Beckman Coulter) and immediately analyzed with a FACSCantoII flow cytometer (BD Bioscience).

### Immunoprecipitation, sodium dodecyl sulfate polyacrylamide gel electrophoresis, and Western blotting

Recombinant human SLC44A2 was produced in Sf9 insect cells transfected with full‐length *SLC44A2* cDNA as described (Kommareddi *et al*, 2009). SLC44A2–NT (CTL2‐NT) rabbit antiserum against synthetic SLC44A2 peptides was coupled to CNBr beads as previously described and used to immunoprecipitate SLC44A2 from cell lysates as described (Nair *et al*, 2004). Whole cell lysates from lung tissues of Slc44a2 wild‐type and knockout FVB mice (Kommareddi *et al*, 2015) were prepared in lysis buffer with 1% NP‐40 and stored frozen until use. UM‐SCC‐47, a human squamous cell carcinoma cell line, naturally expresses wild‐type SLC44A2 and served as a source of wild‐type SLC44A2 protein. Cell lysates of Sf9 insect cells expressing full‐length rHuSLC44A2 and UM‐SCC 47 expressing wild‐type SLC44A2 protein were similarly prepared. The cell lysates (150 μg protein) were immunoprecipitated with anti CTL2‐NT beads at a concentration of 2 μg/ml, collected and washed by centrifugation and subjected to sodium dodecyl sulfate polyacrylamide gel electrophoresis (SDS–PAGE) as described previously (Kommareddi *et al*, 2009). The primary antibodies, rabbit anti‐CTL2‐NT (1/500 or 2 μg/ml), human alloantibodies RIF, and VER were used at 1:10. Antibody binding on Western blots was detected with enhanced chemiluminescence using the appropriate affinity purified secondary antibody. Rabbit antihuman IgG‐IgM‐specific antiserum was diluted 1:2,000. Goat anti‐rabbit IgG heavy and light chain specific was used at 1:5,000.

### Neutrophil and reticulocyte isolation

Human neutrophils were isolated from fresh whole blood (< 4 h after blood sampling) using the MACSxpress Neutrophil Isolation Kit followed by a MACSxpress Erythrocyte Depletion Kit (Miltenyi Biotec). Reticulocytes were isolated from whole blood using the CD71 MicroBeads Kit (Miltenyi Biotec). The purity of the cells, determined by flow cytometry using anti‐CD16 and anti‐CD71 antibodies, was approximately 98%.

### Amplification of *SLC44A2* transcripts

mRNA was extracted from neutrophils and reticulocytes and transcribed with superScript III first‐strand synthesis SuperMix, according to the manufacturer's instructions (Thermo Fisher Scientific). cDNA products were amplified by PCR using a primer combination specific for the P1 and P2 SLC44A2 transcripts. Primer sequences are available in Appendix Table S2.

### Rheology experiments

RBC deformability (EI, elongation index) was determined at 37°C at 9 shear stresses ranging from 0.30 to 30 Pa by laser diffraction analysis (ektacytometry) using the laser‐assisted optical rotational cell analyzer (LORCA, RR Mechatronics).

### Platelet aggregation and activation studies

Platelet aggregation tests in the presence of several agonists were measured with an optical platelet aggregometer (APACT 4004, ELITechGroup, Puteaux, France) according to the manufacturer's instructions. For activation tests, platelets were stimulated or not with different agonists for 15 min and then incubated with various fluorophore‐conjugated antibodies. Anti‐CD62P‐PE was purchased from Becton Biolegend. Anti‐CD41‐PC7 and anti‐CD63‐PE were purchased from Beckman Coulter. Samples were analyzed in an FACSLyric (Becton Dickinson) flow cytometer.

### Flow neutrophil adhesion assays

HMEC‐1 (Human Microvascular Endothelial Cell line) monolayers were grown in Vena8 Endothelial+ Biochips (Cellix Ltd, Dublin, Ireland) as previously described (Koehl *et al*, 2017), and activated by TNFα (10 ng/ml) for 24 h prior to the adhesion assay. In brief, adhesion was measured under flow conditions using Vena8 Endothelial + TM biochips (internal channel dimensions: length 20 mm, width 0.8 mm, height 0.12 mm) and ExiGoTM Nanopumps (Cellix Ltd, Dublin, Ireland). Neutrophils were isolated and incubated with anti‐RIF, anti‐VER, or irrelevant IgG1 antibodies 30 min before cell perfusion. Samples were then perfused for 45 min at 1 dyn/cm^2^ through the biochip channels containing HMEC‐1 monolayers. Adherent neutrophils were labeled with anti‐CD16 alexa 488‐conjugated mouse monoclonal antibody (Biolegend, San Diego, USA) for 15 min. Neutrophil adhesion was monitored using AxioObserver Z1 microscope and ZEN software (Carl Zeiss, Le Pecq, France). Images were taken in 11 representative fields at the centerline of each channel at 10 min intervals throughout the assay. Adhesion levels were quantified by measuring the surface area of fluorescent patches using the ImageJ Software. Adhesion in each channel is defined as the median value of fluorescence of the 11 fields.

### Statistics

Statistical analyses were performed using GraphPad Prism 7. Comparisons between values were performed using Wilcoxon test (nonparametric paired test). The results are expressed as the medians ± standard deviation (SD).

**The paper explained**

**Problem**

Red blood cell (RBC) transfusion is a major therapy in patients' medical care, especially in the treatment of sickle cell disease, malarial anemia, and in hematologic malignancies. Numerous difficulties hamper the optimal use of this vital medical practice, especially the immunological barrier of blood group antigen polymorphisms. However, several antigens have so far eluded discovery of their molecular basis despite intense efforts motivated by the clinical significance of the corresponding antibodies.

**Results**

In this study, we investigated the rare blood phenotypes of individuals with anti‐erythrocyte alloantibodies of unknown specificity. Whole‐exome sequencing (WES) in individuals of Moroccan descent identified a new missense mutation in *SLC44A2*, a gene encoding the choline transporter‐like protein CTL2. We demonstrated that their rare blood group phenotype is underlined by the p.Pro398Thr substitution in SLC44A2. Immunoprecipitation and overexpression experiments confirmed the specificity of alloantibodies and that *SLC44A2* encodes a novel blood group system. Furthermore, we identified three siblings of European ancestry who are homozygous for a large deletion in *SLC44A2*, which results in a null phenotype. Interestingly, the first‐ever reported SLC44A2‐deficient individuals described in this work suffer from progressive hearing impairment, confirming a role of SLC44A2 in hearing maintenance. However, the biological function of this protein in blood cells remains unclear, as SLC44A2_null_ individuals displayed normal *in vitro* erythropoiesis and have no apparent hematological disorders. Since SLC44A2 carries the human neutrophil antigen‐3 (HNA‐3), our findings strengthen the importance of anti‐ SLC44A2 in transfusion medicine.

**Impact**

Overall, we showed that SLC44A2 is a novel blood group system, with direct clinical relevance since this protein deficiency is involved in hearing impairment and possible neurovascular disorders. It is also important in transfusion medicine, with the characterization of two novel blood group antigens, and plasma transfusion of donors with anti‐RIF or anti‐VER can potentially cause a life‐threatening TRALI syndrome, as reported for anti‐HNA‐3a. The characterization of this novel system provides the possibility for developing serological and molecular tools for resolving complex immunohematological cases encountered in patients from North Africa and for a large‐scale screening of rare RIF^−^ blood donors. Such a screening has the potential to reduce the risk of hemolytic transfusion reactions by finding compatible donors for RIF^−^ patients, and the risk of TRALI by excluding plasma donors with an anti‐RIF antibody.

## Author contributions

**Bérengère Koehl:** Investigation; visualization; writing – review and editing. **Cédric Vrignaud:** Investigation; visualization. **Mahmoud Mikdar:** Investigation. **Thankam S Nair:** Investigation. **Lucy Yang:** Investigation. **Seyve Landry:** Investigation. **Guy Laiguillon:** Investigation. **Claudine Giroux‐Lathuile:** Resources. **Sophie Anselme‐Martin:** Resources. **Hanane El Kenz:** Resources. **Olivier Hermine:** Conceptualization; funding acquisition. **Narla Mohandas:** Conceptualization. **Jean Pierre Cartron:** Conceptualization. **Yves Colin:** Conceptualization. **Olivier Detante:** Resources. **Raphaël Marlu:** Resources; investigation. **Caroline Le Van Kim:** Conceptualization. **Thomas E Carey:** Resources; supervision; writing – original draft; writing – review and editing. **Slim Azouzi:** Conceptualization; supervision; funding acquisition; investigation; writing – original draft; project administration; writing – review and editing. **Thierry Peyrard:** Conceptualization; resources; funding acquisition; writing – review and editing.

## Disclosure and competing interests statement

The authors declare that they have no conflict of interest.

## For more information

Information about blood group antigens and transfusion medicine.

- ISBT: https://www.isbtweb.org

Author's homepage

- https://sites.google.com/view/umrs1134-team1

French Blood Establishment

- https://dondesang.efs.sante.fr

## Supporting information



Appendix

Source Data for Figure 3

## Data Availability

The whole‐exome sequencing data for RIF^−^ and VER^−^ negative individuals have been deposited to the SRA (NCBI) with the following ID (PRJNA905421; https://www.ncbi.nlm.nih.gov/bioproject/PRJNA905421).
