# Supplementary material for: Lack of the human choline transporter‐like protein SLC44A2 causes hearing impairment and a rare red blood phenotype
Source: EMBO Mol Med. 2023 Jan 25;15(3):e16320. doi: 10.15252/emmm.202216320 (PMC9994479; doi:10.15252/emmm.202216320)
Supplement: Supplementary file 1 — Appendix [file EMMM-15-e16320-s002.pdf]

Table of contents

Appendix Table S1. Hematological parameters of RIF– and VER– probands (Page 1).

Appendix Table S2. Primers and PCR conditions used to confirm the c.1192C>A mutation by sequencing, the *SLC44A2* deletion breakpoint, the RIF genotyping and analysis of *SLCC4A2* P1 and P2 isoforms (Page 2).

Appendix Figure S1. Pedigree of the family of the RIF-proband indicating the genotype for each individual (Page 3).

Appendix Figure S2. Alignment of the primary amino acid sequences of human and mouse SLC44A2 (CTL2) (Page 4).

Appendix Figure S3. Pedigree of the family of the proband 2 (arrow); solid symbols correspond to VER-negative phenotype (Page 5).

Appendix Figure S4. The elongation index (i.e., deformability) of red blood cells (RBC) from VER proband and healthy control determined at discrete shear stresses between 0.5 and 30.0 Pa (Page 6).

Appendix Figure S5. Expression level of P-selectin (CD62P) and CD63 on platelets from controls and CTL2null patient (IV.5) (Page 7).

Appendix Table S1: Hematological parameters of RIF– and VER– probands.

	RIF–probands (p.Pro398Thr)			VER–probands ($CTL2_{null}$)		
	**1**	**2 (proband 1)**	**3**	**IV.1 (proband 2)**	**IV.5**	**Reference values**
RBCs (10^{12}/L)	3.8	4.8	4.2	4.6	4.7	4-5
HGB (g/dL)	11.4	14.3	13.6	13.3	15	11-15
HCT (%)	35	41.6	39.7	41.1	46.5	35-50
MCV (fL)	90.7	86.7	93.6	91.6	97.7	82-98
MCH (pg)	29.4	29.8	32.1	29	31.5	27-32
MCHC (g/dL)	32.4	34.4	34.3	33.1	32.3	32-35
WBCs (10^9/L)	7.3	6.5	4.4	7.9	7.6	4 -10
Neutrophils (10^9/L)	N.D	N.D	N.D	4.5	N.D	2-8
Platelets (10^9/L)	331	331	157	278	294	150-400
Reticulocytes (10^9/L)	N.D	N.D	N.D	28	N.D	20-150

Appendix Table S2: Primers and PCR conditions used to confirm the c.1192C>A mutation by sequencing, the *SLC44A2* deletion breakpoint, the RIF genotyping and analysis of *SLCC4A2* P1 and P2 isoforms.

	Assay	User Name	Sequence (5' to 3')	Location	Position	Product size
c.1192C>A polymorphism confirmation	PCR	SLC44A2-P1F	CTGTGGGATACGTCATGTGC	Upstream of exon 12	chr19 : 10635165 - 10635184	1221 bp
		SLC44A2-P2R	GTAGAAGGCGAACTGGCAAC	Exon 15	chr19 : 10636385 - 10636366	
	Sequencing	SLC44A2-S1F	AGTGACCTGCAGCTTAGGGA	Upstream of exon 13	chr19 : 10635318 10635337	-
SLC44A2 deletion breackpoint	PCR	SLC44A2-P3F	GGAGTTTCCCAGCCTACCTC	Upstream of 5'UTR	chr19 : 10598153 - 10598172	1331 bp
		SLC44A2-P4R	AACCGTGGGTGGGACGTA	Downstream of exon 16	chr19 : 10636775 - 10636758	
	Sequencing	SLC44A2-P2R	GTAGAAGGCGAACTGGCAAC	Exon 15	chr19 : 10636385 - 10636366	-
RIF Genotyping	ASP-PCR	SLC44A2-P5F	TAAATGGGGGCAGTTGTAGC	Upstream of exon 11	chr19 : 10634679 10634698	821 bp
		SLC44-1192C-R or SLC44-1192A-R	TTCGCAGTAAATGGGCAGGG TTCGCAGTAAATGGGCAGGT	On c.1192C>A polymorphism	chr19 : 10635499 - 10635480	
	Amplification Control	hGH-F	TGCCTTCCCAACCATTCCCTTA	Exon 2 of *hGH* gene	chr17 : 63918421 - 63918442	434 bp
		hGH-R	CCACTCACGGATTTCTGTTGTGTTTC	Exon 3 of *hGH* gene	chr17 : 63918009 63918034	
cDNA analysis	P1 Isoform PCR	SLC44A2-cP1F	ATGGAGGACGAGCGGAAA	Exon 1a	chr19 : 10602531 - 10602548	2291 pb
		SLC44A2-3'UTR	TAAGAAGGAGATGCCCCAGA	3' UTR	chr19 : 10643561 - 10643542	
	P2 Isoform PCR	SLC44A2-cP2F	ATGGGGGACGAGCGGCCCCA	Exon 1b	chr19 : 10625634 - 10625653	2297 pb
		SLC44A2-3'UTR	TAAGAAGGAGATGCCCCAGA	3' UTR	chr19 : 10643561 - 10643542	

SUPPLEMENTAL FIGURES

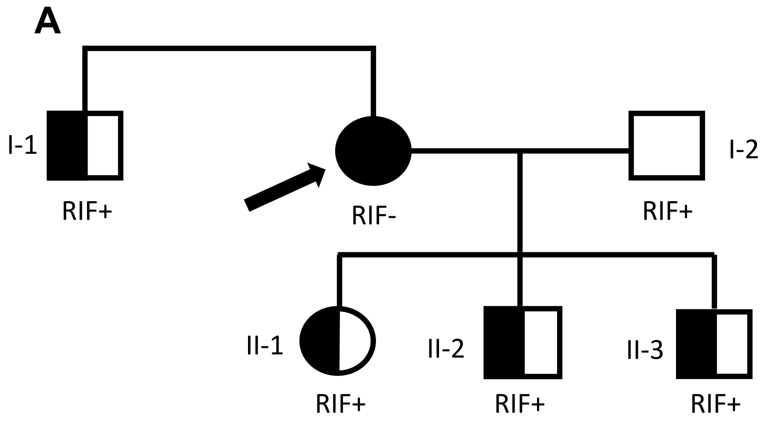

Appendix Figure S1: Pedigree of the family of the RIF-proband indicating the genotype for each individual. Propositus is indicated by an arrow and presents the new mutation in homozygous state.

```
CTL2_HUMAN MEDERKNGAYGTPQKYDPTFKGPIYNRGCTDIICCVFLLLAIVGYVAVGIIAWTHGDPRK 60
CTL2_MOUSE MEDDRKDAVYGTPQKYDPTFKGPIYNRGCTDVICCVLLFLAIVGYVAVGIIAWTHGDPRK 60
           ***:**:..**********************:****:*:*********************

CTL2_HUMAN VIYPTDSRGEFCGQKGTKNENKPYLFYFNIVKCASPLVLLEFQCPTPQICVEKCPDRYLT 120
CTL2_MOUSE VIYPTDSRGEFCGQKGTKNADKPFLFYFNIVKCANPLVLLEFHCPTPQICVKQCPDRYLT 120
           ******************* :**:**********.*******:********::*******

CTL2_HUMAN YLNARSSRDFEYYKQFCVPGFKNNKGVAEVLQDGDCPAVLIPSKPLARRCFPAIHAYKGV 180
CTL2_MOUSE LLSARNTRDFDYYKQFCVPGFQNNKGVTEILRDGECPAVITPSKPLAQRCFPAIHASKGV 180
            *.**.:***:**********:*****:*:*:**:****: ******:******** ***

CTL2_HUMAN LMVGNETTYEDGHGSRKNITDLVEGAKKANGVLEARQLAMRIFEDYTVSWYWIIIGLVIA 240
CTL2_MOUSE LMVGNETTYEDGHGARKNITDLVEGAKKANKILEARQLAMQIFEDYTVSWYWIIIGLVIA 240
           **************:*************** :********:*******************

CTL2_HUMAN MAMSLLFIILLRFLAGIMVWVMIIMVILVLGYGIFHCYMEYSRLRGEAGSDVSLVDLGFQ 300
CTL2_MOUSE MVLSLLFIVLLRFLAGIMVWVMIVMVILVLGYGIFHCYMEYSRLRGEAGSDVSLVDLGFQ 300
           *.:*****:**************:************************************

CTL2_HUMAN TDFRVYLHLRQTWLAFMIILSILEVIIILLLIFLRKRILIAIALIKEASRAVGYVMCSLL 360
CTL2_MOUSE TDLRVYLHLRQTWMAFMIILSILEVVIILLLIFLRKRILIAIALIKEASRAVGHVMCSLL 360
           **:**********:***********:***************************:******

CTL2_HUMAN YPLVTFFLLCLCIAYWASTAVFLSTSNEAVYKIFDDSPCPFTAKTCNPETFPSSNESRQC 420
CTL2_MOUSE YPLVTFFLLCLCIAYWASTSVFLSTSNTAVYKVVDDTACPLLRKTCNPETFPLRNESLQC 420
           *******************:******* ****:.**: **:  *********  *** **

CTL2_HUMAN PNARCQFAFYGGESGYHRALLGLQIFNAFMFFWLANFVLALGQVTLAGAFASYYWALRKP 480
CTL2_MOUSE PTARCQFAFYGGESTYHRALLGLQIFNAFMFFWLANFVLALGQVTLAGAFASYYWAMRKP 480
           *.************ *****************************************:***

CTL2_HUMAN DDLPAFPLFSAFGRALRYHTGSLAFGALILAIVQIIRVILEYLDQRLKAAENKFAKCLMT 540
CTL2_MOUSE DDMPAFPLFSAFGRALRYHTGSLAFGSLILAIVQIIRVMLEYLDQRLKAAQNKFAKFLMV 540
           **:***********************:***********:***********:***** **.

CTL2_HUMAN CLKCCFWCLEKFIKFLNRNAYIMIAIYGTNFCTSARNAFFLLMRNIIRVAVLDKVTDFLF 600
CTL2_MOUSE CLKCCFWCLEKFIKFLNRNAYIMIAIYGTNFCTSARNAFFLLMRNIIRVAVLDKVTDFLF 600
           ************************************************************

CTL2_HUMAN LLGKLLIVGSVGILAFFFFTHRIRIVQDTAPPLNYYWVPILTVIVGSYLIAHGFFSVYGM 660
CTL2_MOUSE LLGKLLIVGSVGILAFFFFTHRIRIVQDTAPPLNYYWVPILTVIIGSYLIAHGFFSVYGM 660
           ********************************************:***************

CTL2_HUMAN CVDTLFLCFLEDLERNDGSAERPYFMSSTLKKLLNKTNKKAAES 704
CTL2_MOUSE CVDTLFLCFLEDLERNDGSAERPYFMSSTLKKLLNKTNKKVAES 704
           ****************************************.***
```

Appendix Figure S2: Alignment of the primary amino acid sequences of human and mouse SLC44A2 (CTL2). The red box corresponds to the amino acid (398Pro) involved in the RIF+/RIF- polymorphism.

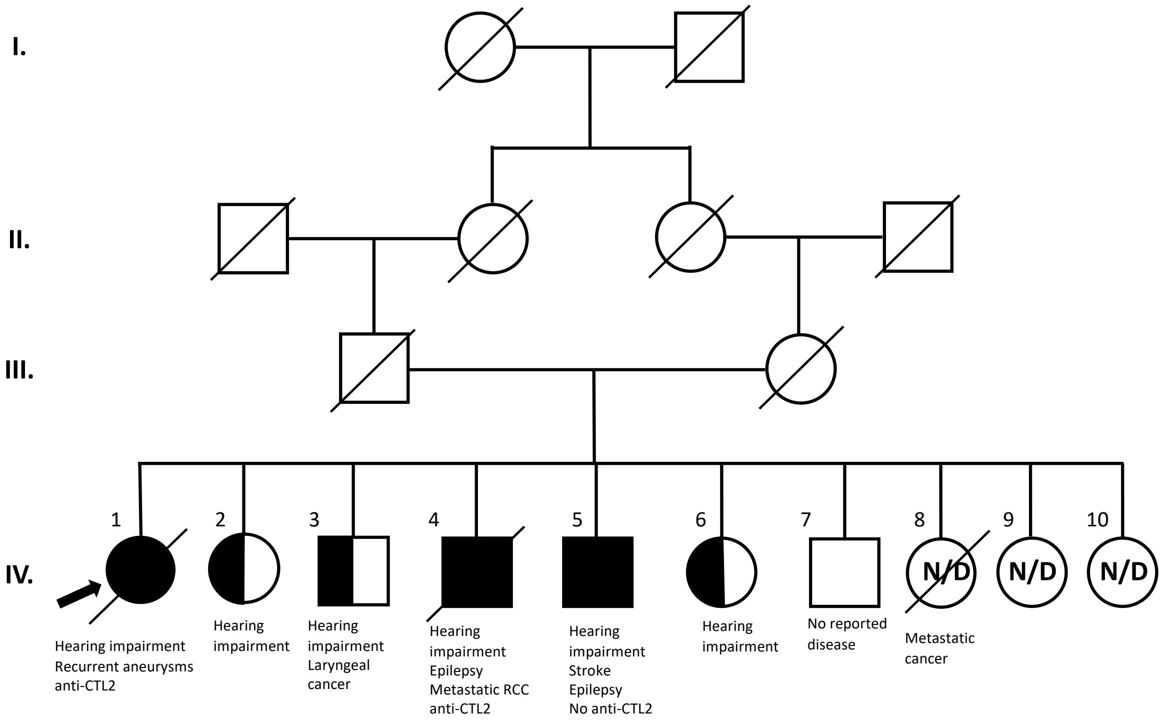

Appendix Figure S3: Pedigree of the family of the proband 2 (arrow); solid symbols correspond to VER-negative phenotype. The half-black symbols indicate heterozygous VER-positive individuals. Open symbol corresponds to homozygous VER-positive individual. N/D, no data concerning the VER phenotype. RCC: Renal cell carcinoma.

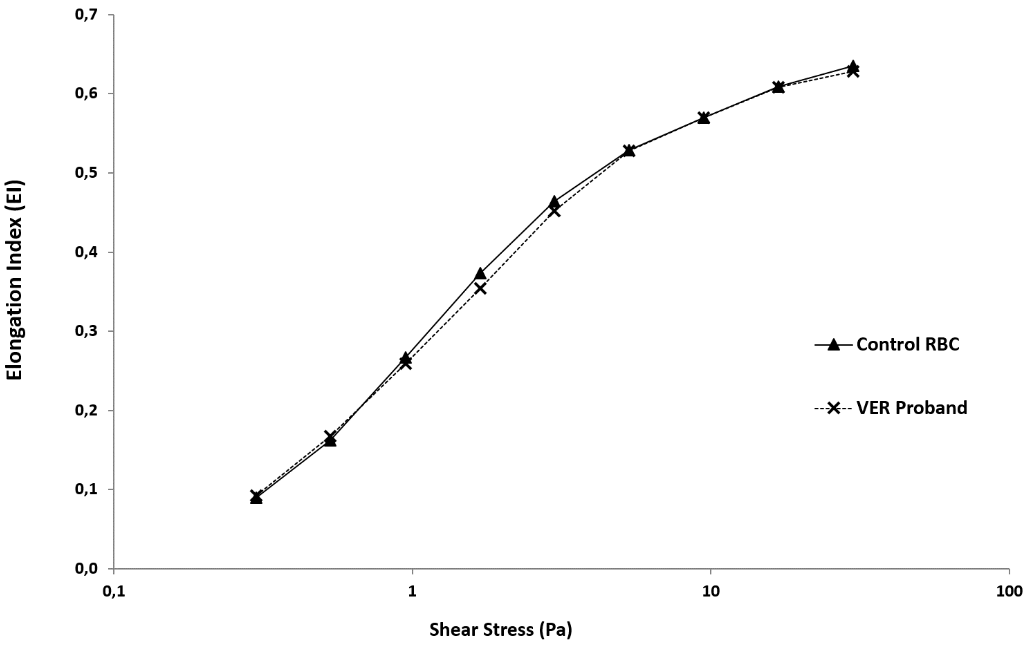

Appendix Figure S4: The elongation index (i.e., deformability) of red blood cells (RBC) from VER proband and healthy control determined at discrete shear stresses between 0.5 and 30.0 Pa.

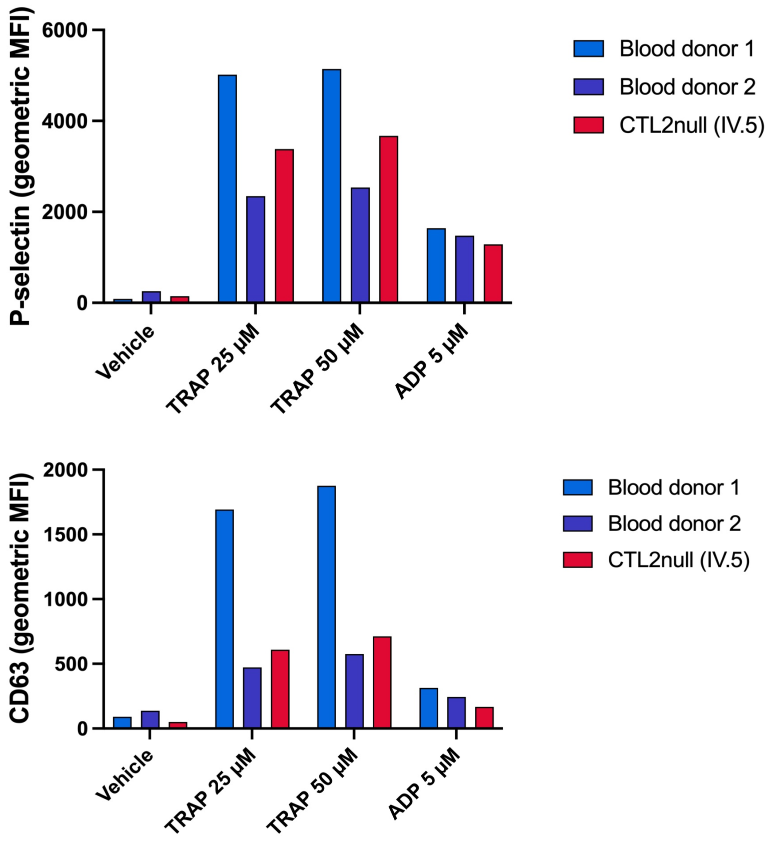

Appendix Figure S5. Expression level of P-selectin (CD62P) and CD63 on platelets from controls and CTL2null patient (IV.5).
