# Supplementary material for: Lack of the human choline transporter‐like protein SLC44A2 causes hearing impairment and a rare red blood phenotype
Source: EMBO Mol Med. 2023 Jan 25;15(3):e16320. doi: 10.15252/emmm.202216320 (PMC9994479; doi:10.15252/emmm.202216320)
Supplement: Supplementary file 2 — Source Data for Figure 3 [file EMMM-15-e16320-s001.pdf]

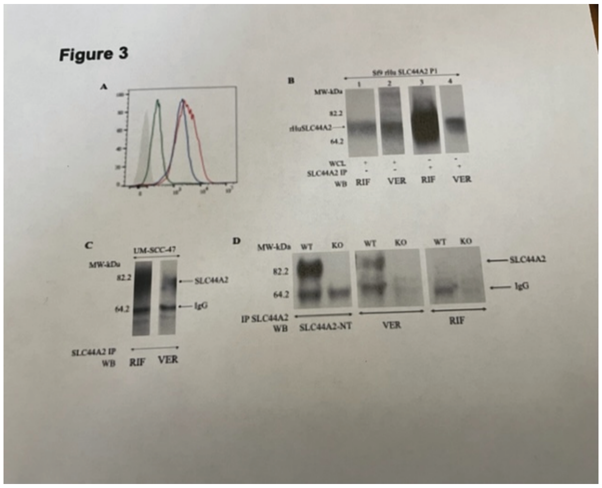

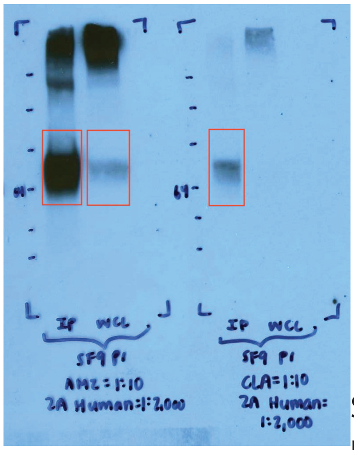

Original for lanes 1-3 fig 3B
10 second exposure

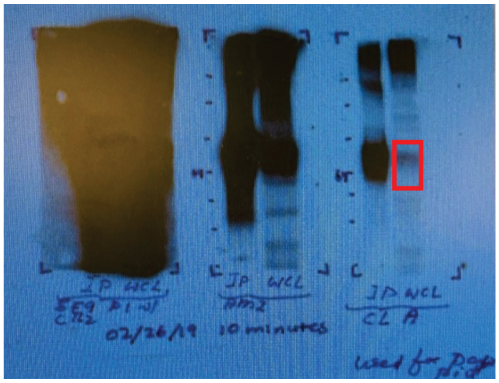

Same blot as left but exposure for 10 minutes showing binding of VER human antibody to whole cell lysate of transfected Insect cells expressing human CTL2whole cell lysate

The lanes in the blots shown above are rearranged in the submitted figure such that the Immuno-precipitated protein lanes are shown in lanes 3 and 4 of the submitted figure. The VER serum (labeled CLA in the blot) has a lower affinity for SCL44A2 protein than the RIF serum (labeled AMZ in the blot).

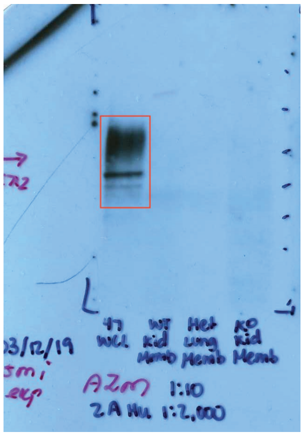

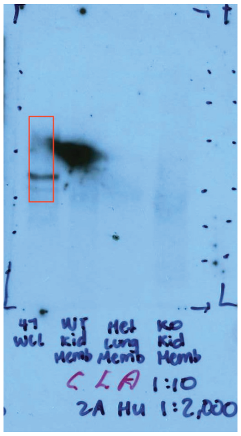

AMZ human Rif serum binding to immunoprecipitate of SLC44a2 (upper band) from human UM-SCC-47 cells. 5 minute exposure.

Human VER (CLA) serum binding to Immunoprecipitated SLC44A2 (upper band) from UM-SCC-47 cells. 5 minute exposure

These images correspond to figure 3c in the submitted MS

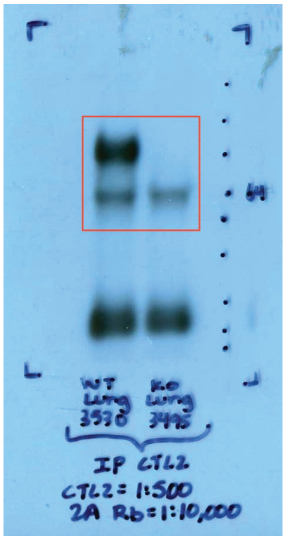

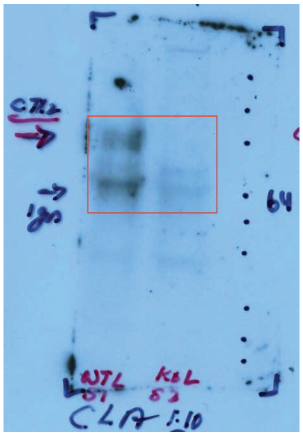

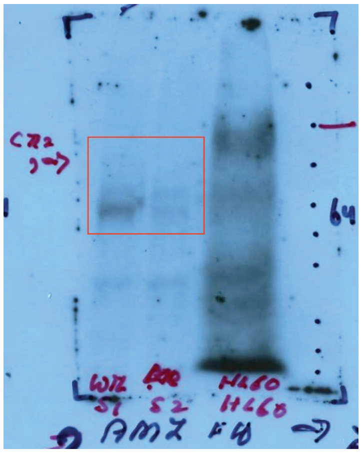

These images are the original blots Used to creat figure 3C.
