# Supplementary material for: Lack of the human choline transporter‐like protein SLC44A2 causes hearing impairment and a rare red blood phenotype
Source: EMBO Mol Med. 2023 Jan 25;15(3):e16320. doi: 10.15252/emmm.202216320 (PMC9994479; doi:10.15252/emmm.202216320)
Supplement: Supplementary file 3 [file EMMM-15-e16320-s003.pdf]

EMBO Molecular Medicine

Lack of the choline transporter-like protein SLC44A2 causes hearing loss and a rare blood phenotype

Berengere Koehl, Cedric Vrignaud, Mahmoud Mikdar, Thankam Nair, Lucy Yang, Seyve Landry, Guy Laiguillon, Claudine Giroux-Lathuile, Sophie Anselme-Martin, Hanane El Kenz, Olivier Hermine, Narla Mohandas, Jean Cartron, Yves Colin, Olivier Detante, Raphaël Marlu, Caroline Le Van Kim, Thomas Carey, Slim Azouzi, and Thierry Peyrard
DOI: 10.15252/emmm.202216320

Corresponding authors: Slim Azouzi (slim.azouzi@inserm.fr) , Thierry Peyrard (thierry.peyrard@efs.sante.fr)

Review Timeline:

Submission Date:	17th May 22
Editorial Decision:	17th Jun 22
Revision Received:	26th Nov 22
Editorial Decision:	23rd Dec 22
Revision Received:	6th Jan 23
Accepted:	10th Jan 23

Editor: Zeljko Durdevic

Transaction Report:

(Note: With the exception of the correction of typographical or spelling errors that could be a source of ambiguity, letters and reports are not edited. Depending on transfer agreements, referee reports obtained elsewhere may or may not be included in this compilation. Referee reports are anonymous unless the Referee chooses to sign their reports.)

17th Jun 2022

Dear Dr. Azouzi,

Thank you for the submission of your manuscript to EMBO Molecular Medicine. We have now received feedback from the three reviewers who agreed to evaluate your manuscript. As you will see from the reports below, the referees acknowledge the interest of the study but also raise important concerns that should be addressed in a major revision.

Further consideration of a revision that addresses reviewers' concerns in full will entail a second round of review. EMBO Molecular Medicine encourages a single round of revision only and therefore, acceptance or rejection of the manuscript will depend on the completeness of your responses included in the next, final version of the manuscript. For this reason, and to save you from any frustrations in the end, I would strongly advise against returning an incomplete revision.

We would welcome the submission of a revised version within three months for further consideration. Please let us know if you require longer to complete the revision.

I look forward to receiving your revised manuscript.

Yours sincerely,

Zeljko Durdevic

Zeljko Durdevic
Editor
EMBO Molecular Medicine

***** Reviewer's comments *****

Referee #1 (Comments on Novelty/Model System for Author):

I would recommend the acceptation of this paper.

Even if I believe that the paper would benefit from a scientific demonstration of the involvement of Slc44a2 in the arterial recurrent aneurysms found in proband 2, I think that the lack of this demonstration does not affect the interest of this work for publication in EMBO molecular medicine.

I would like the authors to do minor changes in phrasing and specially to improve the comparison of their data to already existing studies on Slc44a2.

These data on CT2Lnull patients are very interesting for a better comprehension of this protein in the cardiovascular field.

Referee #1 (Remarks for Author):

In this manuscript, B. Koehl and collaborators describe how investigating alloantibodies to a high prevalence antigen found in individuals led to the identification of a new blood group system. They identify the target antigen (SLC44A2/CTL2) and made some functional work to compare CTL2null platelet function with mouse studies. They also used anti-VER and anti-RIF antibodies to determine their impact on neutrophil activation in flow assays.
The stories leading to the identification of these antibodies, and to the CTL2null proband were of great interest. To correlate the CTL2null human phenotype to SLC44A2-/- mice is also adding interest to this protein. It would have been nice to add some functional studies to associate clinical data on aneurysms (VER- proband 2) to the SLC44A2null phenotype. However, this could be the focus of another work.

I have only minor comments:

(a) The authors state in the abstract and later in the text that they observed normal aggregation of platelets from CTL2null individuals and that these results contrast with what has been previously shown with platelets from SLC44A2-/- mice. However, the only data that are different to already published work are with arachidonic acid and TRAP (Bennett et al., Nat Comm 2020) whereas they are more in accordance with the work published by Tilburg et al., JTH 2020 and Thromb Res 2018, that show no effect of ADP, TRAP-4, thrombin or collagen-related peptide on the aggregation of SLC44A2-/- platelets. Il would be better to compare the authors' results to the different published papers as data existing to date on SLC44A2-/- platelet aggregation studies can be conflicting. I would recommend the authors to reduce the strong statements made for a subject that is in current debate. The authors can avoid such since that does not contribute to the excellent work the authors performed in this manuscript.

(b) Sometimes it is confusing if SLC44A2 and CTL2 are or are not the same protein. It would be nice to have this in the introduction.

(c) Two predominant isoforms of SLC44A2 exist: the P1 and P2 isoforms. The authors have shown that the P2 form is absent from neutrophils and reticulocytes. Others have also observed that P2 is absent from platelets. Only the P2 from can transport choline (Kommareddi et al Prot J 2010). SLC44A2 from platelets is thus not supposed to transport choline. It would be appreciated to have this information in the manuscript as it can be important to evaluate platelet data from Bennett et al, when compared to the data from the reviewed paper.

(d) Do the authors know why the HNA-3a antigen has not been also officialized as blood group antigen of the CTL blood group system?

(e) That would be convenient if the authors could try to give a simple coding system for the reader to exactly know from which proband or which proband's sibling they are talking about all the way throughout the manuscript.

(f) End of page 5: this is not a discrepancy.

(g) page 6: Could the authors correct wording: "This deletion defines a null allele of SLC44A2, explaining why the European proband 2 had developed an alloantibody to SLC44A2/CTL2". This deletion does not allow the identification of the antibody target. Only the figure 3A allows the identification of its target. This statement should arrive after the result.

Referee #2 (Comments on Novelty/Model System for Author):

The technical quality is ranked as medium as some of the approaches could be more thorough. More thorough characterisation of the titre and the regions of SLC44A2 that are targetted would be highly useful.
The use of the isolated antibodies could nicely be used to more thoroughly explore binding to different blood cell types, as well as whether the aggregation induced is caused by the bivalency of the antibody
The identification humans that are deficient in SLC44A2 is highly novel
The medical impact may be comparatively modest given that the incidence of this reains low/very rare.
Gien that the studies are primarily using human samples, this naturally represents an ideal scenario to relate to the human situation

Referee #2 (Remarks for Author):

Overall, the manuscript is potentially interesting. This represents the first report of individuals that are seeingly deficient in SLC44A2.
It remains somewhat unclear why the terminology of RIF and VER were selected. More than anything, I find this confusing given the lack of any obvious meaning to this acronyms - better/more intuitive nomenclature would be favoured
The amino acid sequence presented appears to be incorrect as well as the numbering - the polymorphism is generally accepted to be R154Q (rather than R152Q) - I believe the sequence presented at the start of the signal peptide appears to differ from the sequence reported more widely.
The authors should consider providing more robust characterisation of the platelet function tests that were performmed. The lack of any platelet function deficit is of particular interest given the recent murine data (although in my opinion I favour the conclusion that SLC44A2 is not a major choline transporter in platlets). More thorough presentation of the aggregation data and perhaps also some flow cytometry showing the presentation of P-selectin and activated alpha IIb beta 3 in response to different agonsits would proide a clearer opportunity for the reader to assess the platelet function side off things. The simple presentation of the % aggregation is perhaps inadequate.
The presence of the anti-SLC44A2 alloantibodies are a highly useful tool. The use of these to formally explore the amount of SLC44A2 on the surface of different blood cell would be highly useful. In anti-SLC44A2 induced TRALI, it is clear that the primary effect of the alloantibodies are upon the neutrophils and yet the other cells appear to be untouched. Therefore presentation of the amount of SLC44A2 on the surface (and indeed also the amount of intracellular SLC44A2) would be highly informative and potetnially explain why there is a cell type specific function of the antibodies.
For the neutrophil adhesion studies - could the aggregation and attachment to the endothelium be due to the bivalent nature of the antibodies? Would the same effect be seen using Fab fragments of the antibodies (suggesting that the antibodies are signalling, rather than 'cross-linking' the cells?

Referee #3 (Comments on Novelty/Model System for Author):

I have no issues with this manuscript, only minor suggestions

Referee #3 (Remarks for Author):

Koehl et al. describe a rare blood cell phenotype due to a modified or deficient expression of SLC44A2 protein CTL2 in red blood cells. They identified two new alloantibodies to CTL2 from patients with rare blood types. They named them anti-RIF and anti-VER from African and European patients (probands 1 and 2). The authors sequenced and characterized both sources and established that the RIF phenotype is from SLC44A2 mutant c1192C>A (pPro398Thr) while the VER phenotype is from SCL44A2 gene deletion. The authors provided elegant genetic and protein analyses to demonstrate that anti-RIF and anti-VER

truly target CTL2 protein. They further established differences between the human CTL2null and CTL2 KO mouse and established that CTL2 is not essential for erythroid differentiation but suggested its specific role in hearing and brain vascular function.
Specific comments:
Abstract:
1. I would exclude the first sentence and some related information since this polymorphism was not the subject of this investigation; I would suggest how/why it was possible to identify-develop those two new types of alloantibodies
Results:
2. p5- Suggest why RIF binds to normal but not to mutated protein using the principles of alloantibody development and binding
3. p.6 Does the intragenic deletion of CTL2 produce any protein; did you test with a CTL2 antibody or used only mRNA PCR
4. p.7 The epitope for anti-RIF is assumed to be in the mutated area, but this was not verified, so I would say that other areas of the mouse CTL2 protein could also lack the anti-RIF binding
5. p9. Add reference for CTL2 expression in neutrophils
Discussion
6. Suggest how anti-RIF binds to the normal CTL2 but does not bind to the single-ammino acid mutant CTL2 by using the information from the protein structure
7. Based on the literature for other proteins, suggest why anti-VER was developed by the RIF proband
8. Include more information on TRALI (Transfusion-related acute lung injury) such as its epidemiology and frequency etc.

We would like to thank the reviewers and the Editor for the careful evaluation of our manuscript and for the many useful and constructive comments. We made a concerted effort to address all of the issues raised in the revised manuscript. We preformed additional studies and provided new data from these additional experiments in the revised version. We believe as a result of these efforts the overall quality of our manuscript has significantly improved and our findings have been further strengthened. Detailed responses to Reviewer's comments are outlined below:

***** Reviewer's comments

Referee #1 (Comments on Novelty/Model System for Author): I would recommend the acceptation of this paper.

Even if I believe that the paper would benefit from a scientific demonstration of the involvement of Slc44a2 in the arterial recurrent aneurysms found in proband 2, I think that the lack of this demonstration does not affect the interest of this work for publication in EMBO molecular medicine.
I would like the authors to do minor changes in phrasing and specially to improve the comparison of their data to already existing studies on Slc44a2. These data on CT2Lnull patients are very interesting for a better comprehension of this protein in the cardiovascular field.

Referee #1 (Remarks for Author):

In this manuscript, B. Koehl and collaborators describe how investigating alloantibodies to a high prevalence antigen found in individuals led to the identification of a new blood group system. They identify the target antigen (SLC44A2/CTL2) and made some functional work to compare CTL2null platelet function with mouse studies. They also used anti-VER and anti-RIF antibodies to determine their impact on neutrophil activation in flow assays. The stories leading to the identification of these antibodies, and to the CTL2null proband were of great interest. To correlate the CTL2null human phenotype to SLC44A2-/- mice is also adding interest to this protein. It would have been nice to add some functional studies to associate clinical data on aneurysms (VER- proband 2) to the SLC44A2null phenotype. However, this could be the focus of another work.

We greatly appreciate the reviewer's highly positive feedback on our study. We agree that functional studies would be needed to confirm the role of SLC44A2 in the arterial recurrent aneurysm development. As mentioned by the reviewer, we plan to pursue such efforts in future work.

I have only minor comments:

(a) The authors state in the abstract and later in the text that they observed normal aggregation

of platelets from CTL2null individuals and that these results contrast with what has been previously shown with platelets from SLC44A2-/- mice. However, the only data that are different to already published work are with arachidonic acid and TRAP (Bennett et al., Nat Comm 2020) whereas they are more in accordance with the work published by Tilburg et al., JTH 2020 and Thromb Res 2018, that show no effect of ADP, TRAP-4, thrombin or collagen-related peptide on the aggregation of SLC44A2$^{-/-}$ platelets. Il would be better to compare the authors' results to the different published papers as data existing to date on SLC44A2-/- platelet aggregation studies can be conflicting. I would recommend the authors to reduce the strong statements made for a subject that is in current debate. The authors can avoid such since that does not contribute to the excellent work the authors performed in this manuscript.

We are grateful to the reviewer for this advice. Although our results indicate that the absence of SLC44A2 does not impact platelet function in humans, we agree with the reviewer that in mice models this question remains a subject of debate. We have tempered our statement about the function of Slc44a2 in mice platelets in the Abstract and further elaborated this point in the Discussion section. In addition, as suggested by Reviewer 2, we have performed additional characterization of platelets activation and aggregation from CTL2$_{null}$ patient. Please also see to our response to reviewer 2.

(b) Sometimes it is confusing if SLC44A2 and CTL2 are or are not the same protein. It would be nice to have this in the introduction.
We thank the reviewer for this pertinent comment. We now clearly emphasize this point in the introduction section.

(c) Two predominant isoforms of SLC44A2 exist: the P1 and P2 isoforms. The authors have shown that the P2 form is absent from neutrophils and reticulocytes. Others have also observed that P2 is absent from platelets. Only the P2 from can transport choline (Kommareddi et al Prot J 2010). SLC44A2 from platelets is thus not supposed to transport choline. It would be appreciated to have this information in the manuscript as it can be important to evaluate platelet data from Bennett et al, when compared to the data from the reviewed paper.
Thank you for this very important suggestion. We have now included this important information in the Discussion section.

(d) Do the authors know why the HNA-3a antigen has not been also officialized as blood group antigen of the CTL blood group system?
We thank the reviewer for raising this relevant point.
Anti-HNA-3a/3b alloantibodies probably react with RBCs and likely have a potential of hemagglutination using standard immunohematological methods. However, since the reagent RBCs included in the screening and identification panels are not characterized for their HNA-3a/3b phenotype/genotype, it is virtually impossible to ascertain an anti-HNA-3a or anti-HNA-3b specificity in such a background. Such antibodies would lead to an inconclusive pattern of reactivity and would probably lead to the conclusion (after a comprehensive serological workup) as antibodies of uncommon specificity, with a recommendation for transfusion of RBC units with a non-reactive serological crossmatch at 37°C.
The context was totally different for anti-RIF and anti-VER since they reacted with all reagent RBCs (antibodies to a high-prevalence antigen), with no possibility for selecting negative crossmatched RBC units. This finding prompted us to perform an in-depth investigation of those antibodies and finally led us to characterize the two novel RIF and VER red cell antigens. We speculate that if the red cell antibody identification panels in the future include the HNA-

3a and HNA-3b status of the reagent RBCs, we would probably find out patterns of reactivities consistent with anti-HNA-3a and anti-HNA-3b in patients' plasma. This could then lead to the official recognition of HNA-3a and HNA-3b as additional red cell antigens within the CTL2 system.

(e) That would be convenient if the authors could try to give a simple coding system for the reader to exactly know from which proband or which proband's sibling they are talking about all the way throughout the manuscript.
We thank the reviewer for this suggestion and have now added the family's pedigree of proband 2 (Appendix Figure S3), for improved clarity of the presentation.

(f) End of page 5: this is not a discrepancy.

(g) page 6: Could the authors correct wording: "This deletion defines a null allele of SLC44A2, explaining why the European proband 2 had developed an alloantibody to SLC44A2/CTL2". This deletion does not allow the identification of the antibody target. Only the figure 3A allows the identification of its target. This statement should arrive after the result.

We thank the reviewer for these comments and have made all the suggested changes in the text.
- "To solve this discrepancy" has been replaced by "To further characterize the blood phenotype of this proband".
- We changed "This deletion defines a null allele of SLC44A2, explaining why the European proband 2 had developed an alloantibody to SLC44A2/CTL2" by "which could explain the rare blood phenotype of this proband".

Referee #2 (Comments on Novelty/Model System for Author):

The technical quality is ranked as medium as some of the approaches could be more thorough. More thorough characterisation of the titre and the regions of SLC44A2 that are targetted would be highly useful. The use of the isolated antibodies could nicely be used to more thoroughly explore binding to different blood cell types, as well as whether the aggregation induced is caused by the bivalency of the antibody. The identification humans that are deficient in SLC44A2 is highly novel. The medical impact may be comparatively modest given that the incidence of this reains low/very rare. Gien that the studies are primarily using human samples, this naturally represents an ideal scenario to relate to the human situation.

Referee #2 (Remarks for Author):

Overall, the manuscript is potentially interesting. This represents the first report of individuals that are seeingly deficient in SLC44A2.

It remains somewhat unclear why the terminology of RIF and VER were selected. More than anything, I find this confusing given the lack of any obvious meaning to this acronym - better/more intuitive nomenclature would be favoured.

We thank the reviewer for bringing up these points. The terminologies of RIF and VER antigens were given based on geographical origin of the two probands. RIF stands for the Rif coast area

of Morocco, from where all the patients (or their ancestors) with the p.Pro398Thr change in CTL2 had their origins.
While VER stands for Verona, the city of birth of the proband with the CTL2 null phenotype. Of note, RIF and VER names were officially acknowledged in June 2020 by the Working Party on *Red Cell Immunogenetics and Blood Group Terminology* of the International Society of Blood Transfusion (ISBT). This is the reason we decided to use this official terminology in the manuscript.

The amino acid sequence presented appears to be incorrect as well as the numbering - the polymorphism is generally accepted to be R154Q (rather than R152Q) - I believe the sequence presented at the start of the signal peptide appears to differ from the sequence reported more widely.
We thank the reviewer for bringing up this point. The position of the amino acid associated with the HNA-3a/b depends on the SLC44A2 isoforms, R152 for P1 isoform and R154 for P2 isoform. Since we have shown that only the P1 isoform is expressed in reticulocytes we used the amino acid sequence for this isoform (R152Q). We clarified this point in page 9 "*The human neutrophil antigen 3a (HNA-3a) is located in the first loop of SLC44A2 and is the result of a non-synonymous single nucleotide polymorphism leading to an amino acid exchange p.152Arg>Gln (isoform P1)/p.154 Arg>Gln (isoform P2)*".

The authors should consider providing more robust characterization of the platelet function tests that were performed. The lack of any platelet function deficit is of particular interest given the recent murine data (although in my opinion I favor the conclusion that SLC44A2 is not a major choline transporter in platelets). More thorough presentation of the aggregation data and perhaps also some flow cytometry showing the presentation of P-selectin and activated alpha IIb beta 3 in response to different agonists would provide a clearer opportunity for the reader to assess the platelet function side off things. The simple presentation of the % aggregation is perhaps inadequate.
We totally agree with the comment of the reviewer, and we undertook great effort to mobilize the last SLC44A2null individual still alive for additional platelet investigations. We then performed an additional aggregation test of platelets from the second CTL2null patient. Aggregation parameters for two CTL2null and two healthy donors are presented in the new Table 1.

We also investigated platelet activation in the presence of different agonists by flow cytometry analysis. In contrast to murine study (Bennett et al., Nat Comm 2020), P-selectin expression was not altered in SLC44A2null platelets in the presence of several agonists. Furthermore, CD63 another altered marker in mice study was also not affected in human SLC44A2null platelets. Unfortunately, PAC-1 labeling was not sufficiently effective to conclude regarding activated alpha IIb beta 3 alteration in this patient. These data are now included in appendix Figure S5. We are grateful to the reviewer for helping us clarifying and reinforcing this important point.

The presence of the anti-SLC44A2 alloantibodies are a highly useful tool. The use of these to formally explore the amount of SLC44A2 on the surface of different blood cell would be highly useful. In anti-SLC44A2 induced TRALI, it is clear that the primary effect of the alloantibodies are upon the neutrophils and yet the other cells appear to be untouched. Therefore presentation of the amount of SLC44A2 on the surface (and indeed also the amount of intracellular

SLC44A2) would be highly informative and potentially explain why there is a cell type specific function of the antibodies.

We agree with the reviewer, anti-SLC44A2 alloantibodies are useful tools to investigate CTL2 expression at the cell surface and intracellularly. As shown in the Figure 6C, both alloantibodies showed that SLC44A2 is highly expressed at the neutrophil surface. Unfortunately, since these antibodies are partially purified from the patient's plasma, the amount of antibody generated in these eluates is very low and should be used at ½ dilution for cell labeling, and as such are not suitable for intracellular expression analysis. In addition, most of anti-erythrocyte alloantibodies recognize conformational dependent epitopes which are not conserved on intracellular proteins.

For the neutrophil adhesion studies - could the aggregation and attachment to the endothelium be due to the bivalent nature of the antibodies? Would the same effect be seen using Fab fragments of the antibodies (suggesting that the antibodies are signalling, rather than 'cross-linking' the cells)?

We thank the reviewer for this very relevant comment and the suggestion to use Fab fragments in these experiments. We have attempted to purify these fragments after papain treatment of eluates containing anti-RIF and anti-VER. Unfortunately, we have not detected any reactivity after purification which could be due to the low antibody concentration in the eluate. To address the reviewer's comment on whether the increased neutrophil adhesion to endothelial cells is due to the bivalent nature of the anti-RIF and anti-VER alloantibodies, we performed neutrophil adhesion in the presence of another anti-blood group antigen alloantibody (Anti-EMM) that binds neutrophils (Duval, et al., Blood 2021). As shown below, although anti-RIF induces neutrophil adhesion to endothelial cells, anti-EMM has no effect on neutrophil adhesion, indicating that this effect is not due to the bivalent nature of the antibodies.

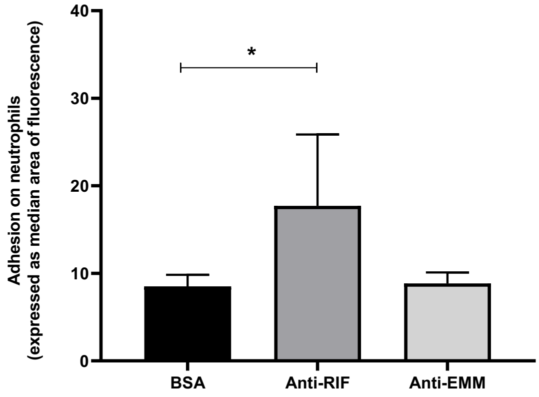

Figure: Anti-RIF red cell alloantibody activates specifically neutrophil adhesion to endothelial cells.

Referee #3 (Comments on Novelty/Model System for Author):

I have no issues with this manuscript, only minor suggestions

Referee #3 (Remarks for Author):

Koehl et al. describe a rare blood cell phenotype due to a modified or deficient expression of

SLC44A2 protein CTL2 in red blood cells. They identified two new alloantibodies to CTL2 from patients with rare blood types. They named them anti-RIF and anti-VER from African and European patients (probands 1 and 2). The authors sequenced and characterized both sources and established that the RIF phenotype is from SLC44A2 mutant c1192C>A (pPro398Thr) while the VER phenotype is from SCL44A2 gene deletion. The authors provided elegant genetic and protein analyses to demonstrate that anti-RIF and anti-VER truly target CTL2 protein. They further established differences between the human CTL2null and CTL2 KO mouse and established that CTL2 is not essential for erythroid differentiation but suggested its specific role in hearing and brain vascular function.

Specific comments:

Abstract:
1. I would exclude the first sentence and some related information since this polymorphism was not the subject of this investigation; I would suggest how/why it was possible to identify-develop those two new types of alloantibodies
We are grateful to the reviewer for this advice. We changed this sentence by *"Blood phenotypes are defined by the presence or absence of specific blood group antigens at the red blood cell (RBC) surface, due to genetic polymorphisms among individuals. The recent devolvement of genomic and proteomic approaches allowed the characterization of several enigmatic antigens."*

Results:
2. p5- Suggest why RIF binds to normal but not to mutated protein using the principles of alloantibody development and binding
As suggested by the reviewer, we included the following sentence in the manuscript: "indicating that Pro398 is important for the expression of RIF antigen and contributes to the epitope recognized by the anti-RIF".

3. p.6 Does the intragenic deletion of CTL2 produce any protein; did you test with a CTL2 antibody or used only mRNA PCR
We have tested two monoclonal anti-human SLC44A2 antibodies (ab115666 and ab57570 from Abcam) but both antibodies failed to detect any specific bands in neither control nor $CTL2_{null}$ RBCs.
Therefore, we only confirmed the absence of CTL2 by RT-PCR in reticulocytes from the CTL2null patient.
4. p.7 The epitope for anti-RIF is assumed to be in the mutated area, but this was not verified, so I would say that other areas of the mouse CTL2 protein could also lack the anti-RIF binding
The 15 exons deleted in *SLC44A2* gene encode the first 410 out of the 704 amino acids of CTL2 protein. RIF antigen is encoded by the Pro398 which is included in the deleted area. We clarified this point by adding this sentence in P.6, "The deleted exons encode the first 410 amino acids of CTL2 protein, which include the Pro398 involved in the expression of RIF antigen."
We agree with the reviewer that other non-conserved amino acids in mouse Slc44a2 could be involved in RIF expression.

5. p9. Add reference for CTL2 expression in neutrophils
This reference has been added.

Discussion

6. Suggest how anti-RIF binds to the normal CTL2 but does not bind to the single-ammino acid mutant CTL2 by using the information from the protein structure.
We thank the Reviewer for this relevant suggestion. We added this paragraph to clarify this point (please refer to page 11): "*The rare RIF– blood type, found in several Moroccan individuals with an anti-RIF alloantibody, is caused by the p. Pro398Thr substitution in SLC44A2 protein. This result indicates that Proline 398, located in the third extracellular loop of SLC44A2 (Figure 6B), encodes the RIF antigen and that Threonine 398 is the molecular basis of the rare RIF– phenotype.*

7. Based on the literature for other proteins, suggest why anti-VER was developed by the RIF proband
The reviewer asked "why anti-VER was developed by the RIF proband". We suppose that the reviewer meant the opposite, i.e. why anti-RIF was developed by the VER– proband? If so, anti-VER developed by VER– individuals can be considered a polyclonal antibody directed to all immunogenic epitopes of the CTL2 protein, including the RIF antigen. We added this paragraph to clarify this point points (please refer to page 11): "*Since the large deletion in SLC44A2 leads to the null VER– phenotype (no CTL2 protein), the RIF antigen is consequently not expressed in VER– RBCs (Figure 2A). Similarly, we have recently demonstrated that the Augustine antigen At^a, carried by the Glutamine 391 in ENT1 nucleoside transporter, is absent from $ENT1_{null}$ RBCs (AUG:-1 phenotype).[7]*"

8. Include more information on TRALI (Transfusion-related acute lung injury) such as its epidemiology and frequency etc.
We added the corresponding paragraph in the Discussion section.

23rd Dec 2022

Dear Dr. Azouzi,

Thank you for the submission of your revised manuscript to EMBO Molecular Medicine. I am pleased to inform you that we will be able to accept your manuscript pending the following final amendments:

1) Please address all referee's points in writing and implement all the suggestions in the manuscript.
2) In the main manuscript file, please do the following:
- In M&M, please include statement that in addition to the WMA Declaration of Helsinki the experiments using human samples also conformed the principles set out in the Department of Health and Human Services Belmont Report.
- In M&M, add statistical paragraph that should reflect all information that you have filled in the Authors Checklist, especially regarding randomization, blinding, replication etc.
3) Synopsis:
- Synopsis image: Please provide the image as a high-resolution jpeg file 550 px-wide x (250-400)-px high.
- Please check your synopsis text and image before submission with your revised manuscript. Please be aware that in the proof stage minor corrections only are allowed (e.g., typos).
4) For more information: This space should be used to list relevant web links for further consultation by our readers. Could you identify some relevant ones and provide such information as well? Some examples are patient associations, relevant databases, OMIM/proteins/genes links, author's websites, etc...
5) As part of the EMBO Publications transparent editorial process initiative (see our Editorial at http://embomolmed.embopress.org/content/2/9/329), EMBO Molecular Medicine will publish online a Review Process File (RPF) to accompany accepted manuscripts. This file will be published in conjunction with your paper and will include the anonymous referee reports, your point-by-point response and all pertinent correspondence relating to the manuscript. Let us know whether you agree with the publication of the RPF and as here, if you want to remove or not any figures from it prior to publication. Please note that the Authors checklist will be published at the end of the RPF.
6) Please provide a point-by-point letter INCLUDING my comments as well as the reviewer's reports and your detailed responses (as Word file).

I look forward to reading a new revised version of your manuscript as soon as possible. Please be aware that due to the holiday season we expect to have a delay in manuscript processing.

Yours sincerely,

Zeljko Durdevic

Zeljko Durdevic
Editor
EMBO Molecular Medicine

F

***** Reviewer's comments *****

Referee #2 (Comments on Novelty/Model System for Author):

The study is not very complex, but does include the highly novel identification of individuals that lack SLC44A2. Given the issues associated with species differences between humans and mice, the identification of these individuals provides highly insightful information with respect to SLC44A2 in humans.

Referee #2 (Remarks for Author):

The strength/importance of the work described in this manuscript is primarily associated with the identification of the individuals that are completely deficient in SLC44A2 - identified via rather fortuitous means. This provides important information on its physiological role of this receptor.
In the introduction, the authors refer to murine SLC44A2 as Slc44a2 - this is correct for the gene, but not for the protein
The authors should check their abbreviations used to ensure everything is appropriately defined (e.g. ISBT)

In all of the flow cytometry figures, it would be highly useful for the authors to provide a color key in the panel to label each lilne and thus avoid continual reference to the figure legend - fig 1c, 2a, 2b, 3a 5a, 6c

In a few locations SLC44A2 and CTL2 are used. Given the gene name, I would use SLC44A2 throughout for consistentcy and to avoid confusion.

The neutrophil agregation assay is a little unusual. It might be more usual to perform these assays with just neutrophils to explore their aggregation induced by the antibodies. It is a little unclear exactly what the authors are measuring. The TNF stimulation of endothelial cells leads to adhesion molecule presentation enabling neutrophiul binindg via a host of receptors (as well as some cell death/detachment. It is thereofre important to understand what the authors are actually measuring in this assay in order to understand it.

Referee #2 (Comments on Novelty/Model System for Author):

The study is not very complex, but does include the highly novel identification of individuals that lack SLC44A2. Given the issues associated with species differences between humans and mice, the identification of these individuals provides highly insightful information with respect to SLC44A2 in humans.
Referee #2 (Remarks for Author):
The strength/importance of the work described in this manuscript is primarily associated with the identification of the individuals that are completely deficient in SLC44A2 - identified via rather fortuitous means. This provides important information on its physiological role of this receptor.
We thank the reviewer for his/her interest in our work. We are also very grateful for the important comments that she/he has raised.

In the introduction, the authors refer to murine SLC44A2 as Slc44a2 - this is correct for the gene, but not for the protein. The authors should check their abbreviations used to ensure everything is appropriately defined (e.g. ISBT).
All these changes are now made in the text.

In all of the flow cytometry figures, it would be highly useful for the authors to provide a color key in the panel to label each line and thus avoid continual reference to the figure legend - fig 1c, 2a, 2b, 3a 5a, 6c

Thank you for this suggestion. We have added a color key in the panel of each figure.

In a few locations SLC44A2 and CTL2 are used. Given the gene name, I would use SLC44A2 throughout for consistency and to avoid confusion.
As suggested by the reviewer, we have changed CTL2 by SLC44A2 throughout.

The neutrophil aggregation assay is a little unusual. It might be more usual to perform these assays with just neutrophils to explore their aggregation induced by the antibodies. It is a little unclear exactly what the authors are measuring. The TNF stimulation of endothelial cells leads to adhesion molecule presentation enabling neutrophil binding via a host of receptors (as well as some cell death/detachment. It is therefore important to understand what the authors are actually measuring in this assay in order to understand it.
We agree with the reviewer and apologize for not making this clearer. Indeed, the flow adhesion assay we used is not an appropriate test to evaluate neutrophils aggregation. In these experiments, we investigated whether anti-RIF and anti-VER are able to increase the neutrophil adhesion to endothelial cells. We quantified the adherent neutrophils by measuring the fluorescence surface using anti-CD16 Alexa 588 labeling. With this approach, the measured fluorescence corresponds to both neutrophil adherent to the endothelial cells and neutrophil aggregates (please see representative fluorescence images below). This has been now clarified in the manuscript (please refer to Page 10 and 11).

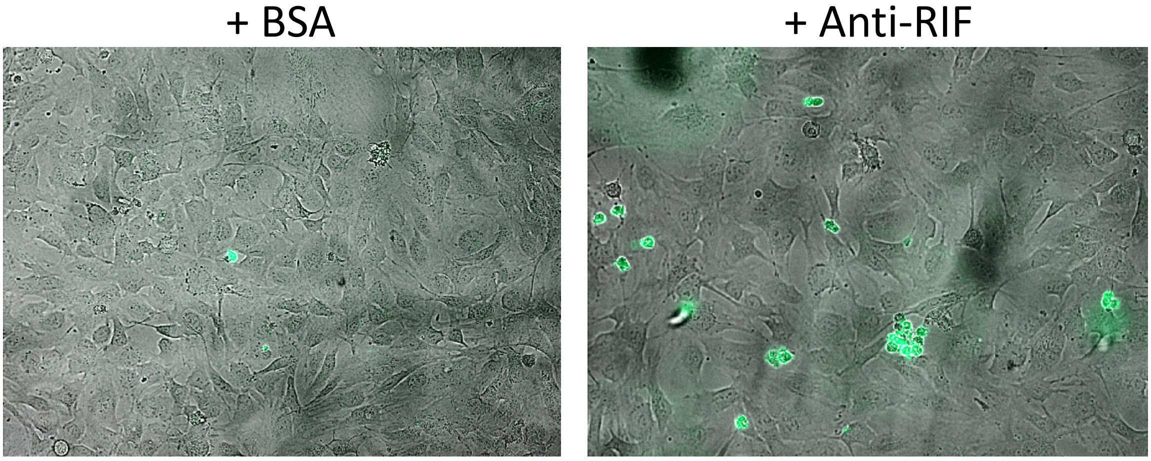

Representative fluorescence images of anti-CD16 Alexa 588-labled untreated neutrophils (+BSA) and treated with anti-RIF.

We are pleased to inform you that your manuscript is accepted for publication and is now being sent to our publisher to be included in the next available issue of EMBO Molecular Medicine.

EMBO Press Author Checklist

Corresponding Author Name: Slim Azouzi and Thierry Peyrard	
Journal Submitted to: EMBO Molecular Medicine	
Manuscript Number: EMM-2022-16320	

USEFUL LINKS FOR COMPLETING THIS FORM
The EMBO Journal - Author Guidelines
EMBO Reports - Author Guidelines
Molecular Systems Biology - Author Guidelines
EMBO Molecular Medicine - Author Guidelines

Reporting Checklist for Life Science Articles (updated January 2022)
This checklist is adapted from Materials Design Analysis Reporting (MDAR) Checklist for Authors. MDAR establishes a minimum set of requirements in transparent reporting in the life sciences (see Statement of Task: 10.31222/osf.io/9sm4x). Please follow the journal's guidelines in preparing your manuscript.
Please note that a copy of this checklist will be published alongside your article.

Abridged guidelines for figures
1. Data
The data shown in figures should satisfy the following conditions:
- the data were obtained and processed according to the field's best practice and are presented to reflect the results of the experiments in an accurate and unbiased manner.
- ideally, figure panels should include only measurements that are directly comparable to each other and obtained with the same assay.
- plots include clearly labeled error bars for independent experiments and sample sizes. Unless justified, error bars should not be shown for technical replicates.
- if n<5, the individual data points from each experiment should be plotted. Any statistical test employed should be justified.
- Source Data should be included to report the data underlying figures according to the guidelines set out in the authorship guidelines on Data Presentation.

2. Captions
Each figure caption should contain the following information, for each panel where they are relevant:
- a specification of the experimental system investigated (eg cell line, species name).
- the assay(s) and method(s) used to carry out the reported observations and measurements.
- an explicit mention of the biological and chemical entity(ies) that are being measured.
- an explicit mention of the biological and chemical entity(ies) that are altered/varied/perturbed in a controlled manner.
- the exact sample size (n) for each experimental group/condition, given as a number, not a range;
- a description of the sample collection allowing the reader to understand whether the samples represent technical or biological replicates (including how many animals, litters, cultures, etc.).
- a statement of how many times the experiment shown was independently replicated in the laboratory.
- definitions of statistical methods and measures:
  - common tests, such as t-test (please specify whether paired vs. unpaired), simple χ2 tests, Wilcoxon and Mann-Whitney tests, can be unambiguously identified by name only, but more complex techniques should be described in the methods section;
  - are tests one-sided or two-sided?
  - are there adjustments for multiple comparisons?
  - exact statistical test results, e.g., P values = x but not P values < x;
  - definition of 'center values' as median or average;
  - definition of error bars as s.d. or s.e.m.

Please complete ALL of the questions below.
Select "Not Applicable" only when the requested information is not relevant for your study.

Materials

Newly Created Materials	Information included in the manuscript?	In which section is the information available? (Reagents and Tools Table, Materials and Methods, Figures, Data Availability Section)
New materials and reagents need to be available; do any restrictions apply?	Not Applicable	

Antibodies	Information included in the manuscript?	In which section is the information available? (Reagents and Tools Table, Materials and Methods, Figures, Data Availability Section)
For **antibodies** provide the following information:<br>- Commercial antibodies: RRID (if possible) or supplier name, catalogue number and or/clone number<br>- Non-commercial: RRID or citation	Yes	Materials and Methods

DNA and RNA sequences	Information included in the manuscript?	In which section is the information available? (Reagents and Tools Table, Materials and Methods, Figures, Data Availability Section)
**Short novel DNA or RNA including primers, probes**: provide the sequences.	Yes	Materials and Methods

Cell materials	Information included in the manuscript?	In which section is the information available? (Reagents and Tools Table, Materials and Methods, Figures, Data Availability Section)
**Cell lines:** Provide species information, strain. Provide accession number in repository **OR** supplier name, catalog number, clone number, and/**OR** RRID.	Yes	Materials and Methods
**Primary cultures:** Provide species, strain, sex of origin, genetic modification status.	Yes	Materials and Methods
Report if the cell lines were recently **authenticated** (e.g., by STR profiling) and tested for mycoplasma contamination.	Not Applicable	

Experimental animals	Information included in the manuscript?	In which section is the information available? (Reagents and Tools Table, Materials and Methods, Figures, Data Availability Section)
**Laboratory animals or Model organisms:** Provide species, strain, sex, age, genetic modification status. Provide accession number in repository **OR** supplier name, catalog number, clone number, **OR** RRID.	Not Applicable	
**Animal observed in or captured from the field:** Provide species, sex, and age where possible.	Not Applicable	
Please detail **housing and husbandry conditions**.	Not Applicable	

Plants and microbes	Information included in the manuscript?	In which section is the information available? (Reagents and Tools Table, Materials and Methods, Figures, Data Availability Section)
**Plants:** provide species and strain, ecotype and cultivar where relevant, unique accession number if available, and source (including location for collected wild specimens).	Not Applicable	
**Microbes:** provide species and strain, unique accession number if available, and source.	Not Applicable	

Human research participants	Information included in the manuscript?	In which section is the information available? (Reagents and Tools Table, Materials and Methods, Figures, Data Availability Section)
If collected and within the bounds of privacy constraints report on age, sex and gender or ethnicity for all study participants.	Yes	Results, Materials and Methods, and Appendix

Core facilities	Information included in the manuscript?	In which section is the information available? (Reagents and Tools Table, Materials and Methods, Figures, Data Availability Section)
If your work benefited from core facilities, was their service mentioned in the acknowledgments section?	Yes	Acknowledgements

Design

Study protocol	Information included in the manuscript?	In which section is the information available? (Reagents and Tools Table, Materials and Methods, Figures, Data Availability Section)
If study protocol has been **pre-registered, provide DOI in the manuscript**. For clinical trials, provide the trial registration number **OR** cite DOI.	Not Applicable	
Report the **clinical trial registration number** (at ClinicalTrials.gov or equivalent), where applicable.	Not Applicable	

Laboratory protocol	Information included in the manuscript?	In which section is the information available? (Reagents and Tools Table, Materials and Methods, Figures, Data Availability Section)
Provide DOI OR other citation details if **external detailed step-by-step protocols** are available.	Not Applicable	

Experimental study design and statistics	Information included in the manuscript?	In which section is the information available? (Reagents and Tools Table, Materials and Methods, Figures, Data Availability Section)
Include a statement about **sample size** estimate even if no statistical methods were used.	Not Applicable	
Were any steps taken to minimize the effects of subjective bias when allocating animals/samples to treatment (e.g. **randomization procedure**)? If yes, have they been described?	Not Applicable	
Include a statement about **blinding** even if no blinding was done.	Not Applicable	
Describe **inclusion/exclusion criteria** if samples or animals were excluded from the analysis. Were the criteria pre-established?<br><br>If sample or data points were omitted from analysis, report if this was due to attrition or intentional exclusion and provide justification.	Not Applicable	
For every figure, are **statistical tests** justified as appropriate? Do the data meet the assumptions of the tests (e.g., normal distribution)? Describe any methods used to assess it. Is there an estimate of variation within each group of data? Is the variance similar between the groups that are being statistically compared?	Yes	Figure captions

Sample definition and in-laboratory replication	Information included in the manuscript?	In which section is the information available? (Reagents and Tools Table, Materials and Methods, Figures, Data Availability Section)
In the figure legends: state number of times the experiment was **replicated** in laboratory.	Yes	
In the figure legends: define whether data describe **technical or biological replicates**.	Yes	

Ethics

Ethics	Information included in the manuscript?	In which section is the information available? (Reagents and Tools Table, Materials and Methods, Figures, Data Availability Section)
Studies involving **human participants**: State details of **authority granting ethics approval** (IRB or equivalent committee(s), provide reference number for approval.	Yes	Materials and Methods
Studies involving **human participants**: Include a statement confirming that **informed consent** was obtained from all subjects and that the experiments conformed to the principles set out in the WMA Declaration of Helsinki and the Department of Health and Human Services Belmont Report.	Yes	Materials and Methods
Studies involving **human participants:** For publication of **patient photos**, include a statement confirming that consent to publish was obtained.	Not Applicable	
Studies involving experimental **animals**: State details of **authority granting ethics approval** (IRB or equivalent committee(s), provide reference number for approval. Include a statement of compliance with ethical regulations.	Not Applicable	
Studies involving **specimen and field samples:** State if relevant **permits** obtained, provide details of authority approving study; if none were required, explain why.	Not Applicable	

Dual Use Research of Concern (DURC)	Information included in the manuscript?	In which section is the information available? (Reagents and Tools Table, Materials and Methods, Figures, Data Availability Section)
Could your study fall under dual use research restrictions? Please check biosecurity documents and list of **select agents and toxins** (CDC): https://www.selectagents.gov/sat/list.htm	Not Applicable	
If you used a select agent, is the security level of the lab appropriate and reported in the manuscript?	Not Applicable	
If a study is subject to dual use research of concern regulations, is the name of the **authority granting approval and reference number** for the regulatory approval provided in the manuscript?	Not Applicable	

Reporting

The MDAR framework recommends adoption of discipline-specific guidelines, established and endorsed through community initiatives. Journals have their own policy about requiring specific guidelines and recommendations to complement MDAR.

Adherence to community standards	Information included in the manuscript?	In which section is the information available? (Reagents and Tools Table, Materials and Methods, Figures, Data Availability Section)
State if relevant guidelines or checklists (e.g., **ICMJE, MIBBI, ARRIVE, PRISMA**) have been followed or provided.	Not Applicable	
For **tumor marker prognostic studies,** we recommend that you follow the **REMARK** reporting guidelines (see link list at top right). See author guidelines, under 'Reporting Guidelines'. Please confirm you have followed these guidelines.	Not Applicable	
For **phase II and III randomized controlled trials**, please refer to the **CONSORT** flow diagram (see link list at top right) and submit the CONSORT checklist (see link list at top right) with your submission. See author guidelines, under 'Reporting Guidelines'. Please confirm you have submitted this list.	Not Applicable	

Data Availability

Data availability	Information included in the manuscript?	In which section is the information available? (Reagents and Tools Table, Materials and Methods, Figures, Data Availability Section)
Have **primary datasets** been deposited according to the journal's guidelines (see 'Data Deposition' section) and the respective accession numbers provided in the Data Availability Section?	Yes	Data availability
Were **human clinical and genomic datasets** deposited in a public access-controlled repository in accordance to ethical obligations to the patients and to the applicable consent agreement?	Not Applicable	
Are **computational models** that are central and integral to a study available without restrictions in a machine-readable form? Were the relevant accession numbers or links provided?	Not Applicable	
If publicly available data were reused, provide the respective **data citations in the reference list**.	Not Applicable	
